# Trophic niche overlap between sympatric harbour seals (*Phoca vitulina*) and grey seals (*Halichoerus grypus*) at the southern limit of their European range (Eastern English Channel)

**DOI:** 10.1002/ece3.7739

**Published:** 2021-07-05

**Authors:** Yann Planque, Jérôme Spitz, Matthieu Authier, Gaël Guillou, Cécile Vincent, Florence Caurant

**Affiliations:** ^1^ Centre d'Études Biologiques de Chizé CEBC, UMR 7372 CNRS/La Rochelle Université La Rochelle France; ^2^ Observatoire Pelagis UMS 3462 CNRS/La Rochelle Université La Rochelle France; ^3^ ADERA Pessac Cedex France; ^4^ Littoral Environnement et Sociétés, LIENSs UMR 7266 CNRS/La Rochelle Université La Rochelle France

**Keywords:** feeding strategies, foraging ecology, intraindividual variability, marine top predators, pinnipeds, stable isotopes

## Abstract

Sympatric harbour (*Phoca vitulina*) and grey seals (*Halichoerus grypus*) are increasingly considered potential competitors, especially since recent local declines in harbour seal numbers while grey seal numbers remained stable or increased at their European core distributions. A better understanding of the interactions between these species is critical for conservation efforts. This study aimed to identify the trophic niche overlap between harbour and grey seals at the southern limit of their European range, in the Baie de Somme (BDS, Eastern English Channel, France), where numbers of resident harbour seals and visiting grey seals are increasing exponentially. Dietary overlap was identified from scat contents using hierarchical clustering. Isotopic niche overlap was quantified using δ^13^C and δ^15^N isotopic values from whiskers of 18 individuals, by estimating isotopic standard ellipses with a novel hierarchical model developed in a Bayesian framework to consider both intraindividual variability and interindividual variability. Foraging areas of these individuals were identified from telemetry data. The three independent approaches provided converging results, revealing a high trophic niche overlap due to consumption of benthic flatfish. Two diet clusters were dominated by either small or large benthic flatfish; these comprised 85.5% [CI95%: 80.3%–90.2%] of harbour seal scats and 46.8% [35.1%–58.4%] of grey seal scats. The narrower isotopic niche of harbour seals was nested within that of grey seals (58.2% [22.7%–100%] overlap). Grey seals with isotopic values similar to harbour seals foraged in coastal waters close to the BDS alike harbour seals did, suggesting the niche overlap may be due to individual grey seal strategies. Our findings therefore provide the basis for potential competition between both species (foraging on benthic flatfish close to the BDS). We suggest that a continued increase in seal numbers and/or a decrease in flatfish supply in this area could cause/amplify competitive interactions and have deleterious effects on harbour seal colonies.

## Graphical abstract

There is a critical need for a better understanding of interactions between two sympatric seal species, the harbour and grey seals, considered as potential competitors. We identified here the overlap in trophic niches between these two species at the southern limit of their European range, in the Baie de Somme (Eastern English Channel, France). All approaches provided converging results and revealed that foraging on benthic flatfish close to the Baie de Somme is the basis for potential competition between harbour and grey seals. We suggest that a continued increase in seal numbers and/or a decrease in flatfish supply in this area could cause/amplify competitive interactions and have deleterious effects on harbour seal colonies.

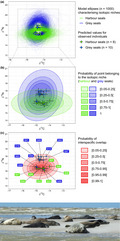

## 1 INTRODUCTION

Detecting interspecific competition between sympatric species is a major objective in ecology as it structures niches and communities (Abrams, 1980; Alley, 1982; MacArthur & Levins, 1967). Competition generally drives the exclusion of less fit species, especially when food resources are limited (Begon et al., 1986; Gause, 1932). Sympatric species with similar functional traits, diets, foraging strategies, and feeding grounds typically present a trophic overlap, and consequently coexist or compete (e.g., Cupples et al., 2011; González‐Solís et al., 1997; Jones & Barmuta, 1998). Since the niche of a species is conceptualized in *n* dimensions defining the resources used in time and space (Hutchinson, 1957), parameters other than diet alone could explain coexistence: foraging on the same prey but at different time periods, and/or at different locations, and/or on different prey sizes/life stages (e.g., Brink et al., 2015). Describing the trophic niches of species in multiple dimensions is therefore necessary to accurately assess potential interactions (Costa‐Pereira et al., 2019; Friedemann et al., 2016).

Directly identifying trophic interactions such as competition in the wild is often impossible, especially when studying highly mobile species. Some studies have succeeded in measuring the effects of interspecific competition directly in the field (e.g., Alatalo et al., 1985; Schoener, 1983). Alatalo et al. (1985) did so on four co‐occurring bird species (*Parus montanus*, *Parus critatus*, *Parus ater*, and *Regulus regulus*) and observed that the foraging niches of the two latter species expanded spatially in testing areas, when the number of *P. montanus* and *P. critatus* were artificially reduced, in comparison with the control area. However, it is complicated (or even impossible) to implement this kind of protocol in many study cases, and thus, measuring trophic niche overlap provides an alternative indirect way to investigate the potential for competition between co‐occurring species (e.g., Ballejo et al., 2018; Ogloff et al., 2019; Pianka, 1974). This is especially true for cryptic and mobile species such as marine top predators which live and feed in a large 3‐dimensional environment wherein experimental setups on a scale commensurate with ecological realism are very limited.

The harbour seal (*Phoca vitulina*) and grey seal (*Halichoerus grypus*) are two sympatric species co‐occurring in the North Atlantic, potentially sharing similar foraging habitats and resources, that make them interesting cases for studying trophic competition. Annual cycles of the two species are asynchronous in European waters as harbour seals breed and molt successively in June–September, while grey seals do so in October–April (Bonner, 1972). Seals increase their time on land and decrease their time at sea during breeding and molting periods, therefore decreasing their foraging activity and restricting feeding to the close vicinity of their haulout sites (Beck et al., 2003; Thompson et al., 1994). The rest of the year they spend most of their time at sea, exhibiting higher foraging activity. Despite a partial trophic segregation in time, they can have similar diets (Thompson et al., 1996; Wilson & Hammond, 2019), diving behavior (Baechler et al., 2002; Lesage et al., 1999; Thompson et al., 1991), and potentially similar foraging grounds in coastal areas (Planque et al., 2020; Thompson et al., 1996). Both species disperse in coastal waters on the continental shelf and can use the same haulout sites (Thompson et al., 1996; Vincent et al., 2017).

Harbour and grey seals are considered generalist feeders at the species level, therefore focusing on prey available locally (Kavanagh et al., 2010; Mohn & Bowen, 1996; Olsen & Bjørge, 1995). Harbour seals restrict their foraging effort to narrower spatial areas, generally in the vicinity of their haulout sites (e.g., Thompson et al., 1996; Vincent et al., 2017), while grey seals travel much further to foraging grounds. Harbour seal diets are highly variable between sites (e.g., Olsen & Bjørge, 1995; Spitz et al., 2010), probably due to differences in prey available locally, with lower variability within a site (e.g., Spitz et al., 2015). Spitz et al. (2010) found harbour seal diets to be homogeneous at the scale of a colony, but later found substantial differences between two colonies located in two different bays (separated by ∼200 km in the Eastern English Channel) with very similar ecological characteristics and fish assemblages (Spitz et al., 2015), suggesting that local foraging habits may exist in harbour seal colonies and that foraging strategies may be passed onto pups by parental and alloparental investments. Thus, there would be more similarity in foraging behavior (including diet and foraging areas) between harbour seal individuals than there is between grey seal individuals. The higher degree of individual specialization in grey seals can be seen in their diet (Tucker et al., 2008), foraging patterns from carbon and nitrogen stable isotopes (Hernandez et al., 2019; Tucker et al., 2007), and diversity of foraging areas (Austin et al., 2004). This specialization could arise from the ontogeny of foraging behavior during early life at sea, in the absence of parental postweaning investment and teaching (Carter et al., 2017).

Drastic declines in harbour seal numbers have been locally observed along the western and eastern Atlantic coasts over the last decades, and trophic competition with the increasing number of grey seals has been suggested as a potential cause (Bowen et al., 2003; Hanson et al., 2013; Jones et al., 2015; Sharples et al., 2012; Svensson, 2012; Thompson et al., 2019). Grey seal predation on harbour seals has also recently been observed (van Neer et al., 2015), but the extent of these direct interactions and their effects on populations is poorly documented. Understanding competitive interactions between these two species is therefore a key objective in the study of their ecology (Bowen et al., 2003; Damseaux et al., 2021; Wilson & Hammond, 2019) and ultimately the management of these populations. In their European core distribution, Wilson and Hammond (2019) found that harbour seals declined in Great Britain where sandeels (Ammodytidae), previously identified as a key prey species in both harbour and grey seal diets, also declined. This suggested that sandeel stock depletion in the North Sea could have had a trophic effect on interspecific competition. However, sandeels were still abundant in grey seal diet after these declines, and thus, Wilson and Hammond (2019) suggested that competition for this prey type might have caused deleterious effects in some harbour seal colonies, affecting their population dynamics. Recent results in the North Sea suggest that the trophic niches of the two species are now mostly segregated due to the continued consumption of sandeels by grey seals and a wider range of other prey types by harbour seals (Damseaux et al., 2021).

**FIGURE 1:**
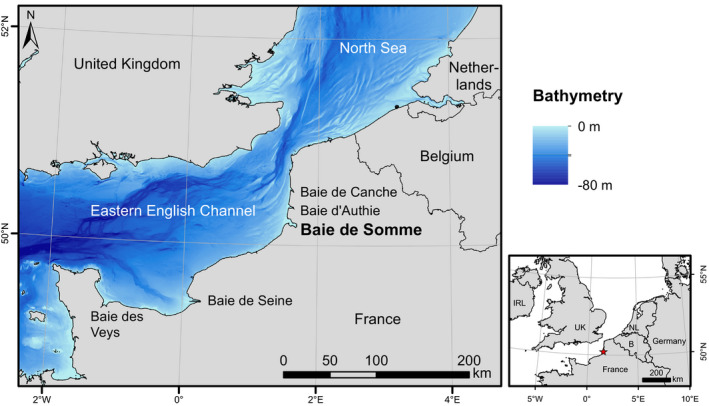
Location of the Baie de Somme (along the French coast of the Eastern English Channel) used by harbour and grey seals as a haulout site, and where individuals were captured and tagged in 2008 and 2012 for stable isotope analyses and foraging areas identification. Bathymetry data were obtained from SHOM (2015)

Populations living at the extent of the species range are particularly interesting cases to study ecological processes as animals evolve in environmental conditions that are more limiting compared with the core distribution (Brown et al., 1996; Sexton et al., 2009). Processes including trophic competition could therefore be exacerbated in such areas. The southern limit of harbour and grey seals' European range is located along the French coast of the English Channel (Vincent et al., 2017), where the main sympatric haulout site of both species is the Baie de Somme (BDS; Eastern English Channel—EEC; location in Figure 1). Harbour seals recolonized this area in the 1990s and have since established a breeding colony (139 pups in 2018; Poncet et al., 2019). This relatively new harbour seal colony still shows dynamic changes in its size and structure, evidenced by the high proportion of subadults (estimated at around 57% of individuals, with data from 2011 to 2014; Vincent et al., 2018). Individual grey seals from the North Sea arrived in the EEC in the 2000s but do not breed in this area (Vincent et al., 2017), which is reflected in the unbalanced sex ratio with a high dominance of adult males (estimated at around 81% of individuals in the BDS; Vincent et al., 2018). The number of individuals of both species is low in this area (maximum yearly counts of 621 harbour seals and 269 grey seals in the BDS in summer 2018; Poncet et al., 2019) compared with the core distribution (e.g., 45,100 [CI95%: 37,000–60,400] harbour seals and 150,000 [131,000–171,600] grey seals in the nearby United Kingdom in 2017; SCOS, 2018). Seal numbers are increasing exponentially in the BDS for both species, faster for grey seals (+21.4% per year) than for harbour seals (+14.6% per year) (Vincent et al., 2017). Diets of harbour seals in this area are essentially composed of small flatfish from nurseries in the summer period (Spitz et al., 2015), and their foraging areas are very coastal and close to the BDS haulout site (Planque et al., 2020). Grey seal foraging areas include these coastal areas as well as areas further offshore (Planque et al., 2020).

While harbour and grey seal numbers are still increasing at the southern limit of their European range, the level of interspecific competition (if there is any) may still be too low to impact seal population dynamics in this area. Resources used by seals may not currently be limited; however, they may become limited in the short‐ or mid‐term if fish stocks decline and functional reorganization of fish communities observed in the BDS these last three decades continue (Auber et al., 2017; McLean et al., 2019). This study therefore aims to identify the trophic niche overlap between harbour and grey seals at the southern limit of their European range, in the BDS (France), in order to identify if there is potential for trophic competition, and, if so, what the associated foraging strategy (or strategies) could be. We used complementary analyses to measure the overlap, considering several dimensions of trophic niches. The diets of both seal species were identified from scat contents and compared in order to document prey types that could cause an overlap. We analyzed the carbon and nitrogen stable isotopes (δ^13^C and δ^15^N) in seal whiskers to define isotopic niches at a colony level and quantify their overlap. Finally, we identified the foraging areas of individuals based on telemetry data in order to specify where the overlap could occur, and to determine if the overlap may be due to individual cases by looking at individuals' stable isotope values. Identifying the potential for competition between sympatric harbour and grey seals at their range limit, where resources are potentially limited, would allow us to describe the nature of competitive interactions (i.e., implicated time periods, prey types, spatial location), and potentially anticipate, and hopefully mitigate, the impacts they could have on population dynamics in this area.

## 2 MATERIAL AND METHODS

### 2.1 Study area

This study was conducted in the Baie de Somme (BDS), a macrotidal estuary located on the French coast of the Eastern English Channel (EEC) (Figure 1). The EEC is characterized by very shallow waters on the continental shelf (mostly shallower than 50 m), strong tidal currents (e.g., Sentchev & Yaremchuk, 2007), and ecosystems strongly structured by the presence of several highly productive estuaries (Baie de Seine, Baie de Somme BDS, Baie d'Authie, Baie de Canche, Baie des Veys; e.g., Carpentier et al., 2009; Girardin et al., 2018; Riou et al., 2001). The BDS estuary is the second most significant fish nursery ground of the EEC, after the Baie de Seine, and is especially important for commercial flatfish species (mainly *Solea solea* and *Pleuronectes platessa*; Carpentier et al., 2009; Riou et al., 2001; Selleslagh et al., 2009).

### 2.2 Diet analysis

Harbour and grey seal diets were analyzed based on the hard prey remains contained in scat samples collected in the BDS from 2002 to 2019 (Table 1). Scat samples were stored frozen at −20℃ until laboratory analyses. The seal species that produced each scat sample was assigned using the DNA analysis described by Spitz et al. (2015). Of the total number of scats collected, 193 harbour seal and 77 grey seal scats contained at least one measurable prey (i.e., detected with diagnostic hard parts) and were included in this study (three harbour seal and 49 grey seal scats were empty of diagnostic hard parts and were therefore excluded) (Table 1). We distinguished two seasonal periods for seal diet analysis: spring/summer (from April to September) and autumn/winter (from October to March). A portion of harbour seal scats included in this study (83 out of 193) were analyzed by Spitz et al. (2015).

**TABLE 1:** Collection of harbour and grey seal scat samples in the Baie de Somme from 2002 to 2019

Seal species	Sampling period	Number of non‐empty scat samples		
		Spring/Summer April–September	Autumn/Winter October–March	Total
Harbour seals	2002–2019	182 (102)	11 (8)	193 (110)
Grey seals	2017–2019	23 (23)	54 (54)	77 (77)

Note

Only non‐empty scat samples are presented here. The number of new scats analyzed in addition to those presented in Spitz et al. (2015) is in brackets.

Diet analysis followed a usual procedure for pinnipeds (Pierce & Boyle, 1991; Ridoux et al., 2007; Spitz et al., 2010). Scat samples were washed on a 0.2‐mm mesh sieve in order to analyze the diagnostic hard remains, such as fish otoliths, fish bones, and cephalopod beaks. These items were identified to the species level using available keys and guides (Härkönen, 1986; Tuset et al., 2008) as well as our own reference material. Taxonomic identification of prey was done to species, group of species pooled, or family level.

Harbour and grey seal diets are presented in terms of the proportion of reconstructed mass of ingested prey per seal species (i.e., all samples pooled for a species), sometimes grouped by season/year. We measured the length or width of fish otoliths, according to fish species or group of fish species, and the lower rostral length of cephalopod beaks. These measurements were then converted into individual body length and body mass using available allometric relationships (Brown & Pierce, 1998; Clarke, 1986; Coull et al., 1989; Härkönen, 1986; Leopold et al., 2001; Lundström et al., 2007; *Observatoire Pelagis* unpublished data; used relationships and the associated source are presented in Appendix 1). We therefore reconstructed the prey body mass associated with each measurable (i.e., not broken or not too much eroded) fish otolith or lower cephalopod beak. A prey species or group was considered present in a sample when at least one diagnostic part was found. The number of prey individuals in a scat sample was estimated from the total number of diagnostic hard parts, including those that were broken or not measurable. The total number of fish individuals per scat sample was estimated as either half the number of paired structures (e.g., otoliths, operculum, dentary, premaxillary bones) rounded up to the nearest integer, or the total number of unpaired structures (e.g., parasphenoid), whichever was higher. The number of cephalopods was estimated as the highest number of either upper or lower beak. The reconstructed body masses of prey individuals were added up to get the relative reconstituted mass for each prey taxon in a scat sample. When the total number of individuals (identified using all diagnostic hard parts) for a given species was higher than the number of individuals with measurable parts, we used the average body mass of measured individuals to reconstruct the total ingested mass at the species level. The degree of digestive erosion of each otolith was not noted in this study, and thus, digestion correction factors (DCFs) could not be applied on these items. Prey body size and mass calculated here could therefore be underestimated from the absolute/real size and mass of prey ingested.

Harbour and grey seal diets were characterized using prey functional groups rather than classic taxonomic groups as they are more descriptive of predator–prey interactions (Smith et al., 2015; Spitz et al., 2018) and more helpful for identifying associated foraging strategies. Prey species (or groups of species) with similar functional traits were pooled into six functional groups (Table 2): small benthic flatfish, large benthic flatfish, benthic non‐flatfish, demersal fish, pelagic fish, and pelagic squids. Benthic flatfish were divided into two functional groups of small and large benthic flatfish, based on the substantial ecological differences between juvenile flatfish and mature/adult flatfish despite being taxonomically associated: juvenile flatfish are dependent on nursery grounds in estuarine and coastal waters during their early life stage (e.g., about 2 years for *S. solea* in the EEC) and are then recruited to the adult stage during which they move to deeper offshore waters to forage (e.g., Riou et al., 2001; Rochette et al., 2010). Benthic flatfish with relative body lengths greater than or equal to 200 mm were considered as large, and anything below that was considered small.

**TABLE 2:** Measurable prey observed in harbour and grey seal scats, and associated functional groups

Family	Prey	Functional group
Pleuronectidae	*Pleuronectes platessa*/*Platichthys flesus*	Small benthic flatfish (relative length <200 mm) or Large benthic flatfish (relative length ≥200 mm)
Soleidae	*Solea solea*/*Pegusa lascaris*	
	*Buglossidium luteum*/*Microchirus variegatus*	
Bothidae	Und. Bothidea	
	*Scophtalmus* spp.	
	*Phrynorhombus norvegicus*	
Gobidae	Und. Gobidae	Benthic non‐flatfish
Trachinidae	*Trachinus draco*	
Callionymidae	*Callionymus lyra*	
Triglidae	Und. Triglidae	
Gadidae	*Merlangius merlangus*	Demersal fish
	*Trisopterus* spp.	
	*Gadus morhua*/*Pollachius* spp./*Melanogrammus aeglefinus*	
Ammodytidae	Und. Sandeels	
Moronidea	*Dicentrarchus labrax*	
Mugilidae	Und. Mullets	
Clupeidea	*Clupea harengus*	Pelagic fish
	*Sardina pilchardus*	
	*Sprattus sprattus*	
Belonidae	*Belone belone*	
Carangidae	*Trachurus trachurus*	
Loliginidae	*Loligo* spp.	Pelagic squids

Note

Und.: undetermined species.

Diet data were set in a two‐dimensional matrix of proportion of total reconstructed prey mass summed by functional group (six columns) for each seal scat sample (270 lines). It was constructed on all non‐empty scats without prior distinction between the two seal species. Diet matrix was scaled with the function “scale” from the *base* in R (version 4.0.2, R Core Team, 2020) to perform an agglomerative hierarchical cluster analysis. This cluster analysis grouped seal scats that had a similar prey composition. The hierarchical cluster analysis was performed using a Euclidian distance procedure to estimate similarity between scats and employed the Ward.D2 algorithm to gather scats in groups (Murtagh & Legendre, 2014). The optimal number of clusters was determined using the “NbClust” function from the *NbClust* package (version 3.0) in R. The selected number of clusters is based on the most popular suggestion resulting from running 30 different indices (more details in Charrad et al., 2014). Cluster analysis was run using the “clustCoDa” function in the *robCompositions* package (version 2.3.0) in R.

The functional composition of the diet associated with each cluster was presented by calculating the percentage of total prey mass of all scats within that cluster by functional group for each seal species separately. Confidence intervals (CI95%) around these percentages by mass were generated for each prey functional group with a bootstrap procedure (Reynolds & Aebischer, 1991) using custom R code. All N seal scats associated with a cluster were randomly resampled with replacement N times to study their overall composition of prey (in mass), and the procedure was repeated 1,000 times to generate a set of 1,000 sampled diet compositions. The lower and upper bounds of the CI95% of diet composition associated with each cluster were defined as the quantiles at 2.5% and 97.5% of the values generated.

### 2.3 Dietary niche characteristics and interspecific overlap

Interspecific dietary overlap between harbour and grey seals was quantified by comparing the functional group composition of their diets in prey mass, without consideration for diet clusters, with the Pianka index (Pianka, 1974) indicated here by O. The index ranges from 0 to 1, where 0 indicates no overlap and 1 indicates a complete overlap; segregation was considered substantial when Pianka values were <0.4 (Ross, 1986):

$$O=\frac{\sum PiAPiB}{\sqrt{\sum PiA^{2}\sum PiB^{2}}}$$

where PiA is the percentage by mass of prey in the functional group i found in harbour seal diets and PiB is the percentage by mass of prey in the functional group i found in grey seal diets. Confidence intervals (CI95%) around the Pianka value were estimated by randomly comparing the two sets of 1,000 sample diet compositions 10,000 times.

### 2.4 Stable isotope analyses

Stable isotope analyses were conducted on the longest whiskers plucked from eight harbour seals and ten grey seals captured in the BDS in 2008 and 2012, respectively (Table 3). Whisker sampling was done during fieldwork that included deployment of tracking devices (cf. information in Section 2.7).

**TABLE 3:** Details of the eight harbour seals and ten grey seals captured in the Baie de Somme, in 2008 and 2012, respectively, each was fitted with GPS/GSM tags for telemetry tracking and had their longest whisker sampled to analyze the δ^13^C and δ^15^N stable isotope composition

Seal species	Individual	Sex	Body mass kg	Body length cm	Telemetry tracking with GPS/GSM tags			δ^13^C and δ^15^N stable isotope analyses on whisker segments	
					Capture date/Start tracking	End tracking	Tracking duration days	Whisker length mm	Number of whisker segments
Harbour seals	S01	M	86	141	2008‐10‐20	2009‐01‐13	85	65	7
	S02	M	82	151	2008‐10‐20	2009‐05‐04	196	103	10
	S03	M	85	139	2008‐10‐20	2009‐04‐03	165	88	10
	S04	M	55	130	2008‐10‐21	2009‐02‐19	121	109	11
	S05	M	89	140	2008‐10‐21	2009‐01‐28	99	102	11
	S06	F	77	143	2008‐10‐21	2009‐01‐03	74	62	7
	S07	M	98	144	2008‐10‐21	2009‐05‐05	196	116	13
	S08	M	83	138	2008‐10‐21	2009‐05‐05	196	90	10
Grey seals	G01	M	98	148	2012‐05‐29	2012‐10‐30	154	82	9
	G02	M	150	188	2012‐05‐30	2012‐10‐20	143	72	8
	G03	M	112	162	2012‐05‐30	2012‐12‐16	200	114	12
	G04	M	146	173	2012‐05‐30	2012‐11‐30	184	72	8
	G05	M	86	149	2012‐05‐30	2012‐11‐03	157	110	12
	G07	M	180	202	2012‐05‐31	2012‐09‐03	95	52	6
	G08	M	139	183	2012‐05‐31	2013‐01‐09	223	100	11
	G09	M	61	140	2012‐06‐01	Tag malfunction		132	14
	G10	M	70	149	2012‐09‐10	2012‐10‐04	24	102	11
	G12	M	~200	205	2012‐09‐13	2013‐01‐02	111	110	12

Note

Body length was measured nose to tail. Stable isotope analyses were performed on whisker segments around every 10 mm.

Seal whiskers are composed of inert keratinous tissue and provide a temporal integration of isotopic information during the period of whisker growth (Hirons et al., 2001; Hobson et al., 1996; Zhao & Schell, 2004). The longest whisker was collected on each individual in order to provide the longest time integration in the stable isotope analysis. Average whisker length was 92 ± 20 mm for harbour seals and 95 ± 24 mm for grey seals (Table 3). The growth of seal whiskers is known to be asymptotic and possibly irregular over months depending on the species and their biology (Greaves et al., 2004; McHuron et al., 2016, 2020; Zhao & Schell, 2004). Harbour seal whisker growth is not continuous over time and shows seasonal variations: for a new whisker (if the preceding one was shed in May/June), the growth rate is high in summer and autumn (0.78 mm per day) and much lower in winter and early spring (around 0.075 mm per day; as estimated from December to May on a captive harbour seal) (Zhao & Schell, 2004). We can therefore assume that the longest harbour seal whisker collected in October 2008 may have incorporated prey foraged at least in the 4 months preceding, i.e., late spring, summer, and early autumn of that year. Grey seal whisker growth is estimated to be around 0.24 mm per day over several months, but with no clear seasonal trends (Greaves et al., 2004). Because this growth is asynchronous and not continuous for this species, we cannot estimate the potential dates associated with a stable isotope measure along the whisker. However, regarding the general growth over months, we can estimate that grey seal whiskers of around 100 mm collected in May 2012 could have been growing for over a year, i.e., at least since May 2011. A whisker collected in September 2012 may have been growing at least since September 2011. Most of the grey seal whiskers we studied therefore provide information on foraging patterns for almost all seasons, including summer, except for the shortest individual whisker (G07, 52 mm). These previous estimations may be very approximative as there is still a lack of recent and more accurate knowledge on the growth patterns of harbour and grey seal whiskers, and must therefore be treated with caution. The retention time of phocid seal whiskers is usually assumed to be annual with a loss during the molting period, but recent studies on some phocid species (other than harbour and grey seals) also highlighted a biennial retention of some whiskers (e.g., Beltran et al., 2015; McHuron et al., 2016). Supposing that some harbour and grey seal whiskers could also be characterized by a biennial retention, we cannot exclude that some of the whiskers used here have grown during longer periods (i.e., up to 2 years).

All samples were cleaned before performing stable isotope analysis in order to remove impurities which could bias isotopic measurements. Each whisker was individually soaked in a bath of 100% ethanol, and impurities were removed by cleaning it manually. The samples were then successively soaked in three different beakers of Milli‐Q ultrapure quality water. Then, they were set up in a beaker of Milli‐Q ultrapure quality water placed in an ultrasonic bath for 20 min. The whiskers were finally placed in an oven at 50℃ for 24 hr. After being washed and dried, each whisker was sectioned into approximately 2 mm sections from the proximal to the distal end. Each section was identified with a reference corresponding to the individual sampled and the distance from the whisker base (i.e., the proximal end; in mm). Finally, for each whisker, we took a section each 10 mm, excluding the first section that was in the seal skin, and these sections were then sent for carbon and nitrogen stable isotope analysis.

All whisker sections were analyzed with an elemental analyzer (Flash 2000, Thermo Scientific) coupled to an isotope‐ratio mass spectrometer (Delta V Plus with a Conflo IV interface, Thermo Scientific). Results were expressed with the usual δ notation in parts per thousand (‰) relative to the Vienna PeeDee Belemnite Standard for δ^13^C and atmospheric N_2_ for δ^15^N. Based on replicate measurements of USGS‐61 and USGS‐62 used as laboratory internal standards, experimental analytical precision was <0.10‰ for δ^13^C and <0.15‰ for δ^15^N. Raw isotopic data measured along each whisker are presented in Appendix 2.

Harbour and grey seal isotopic niches in δ^13^C and δ^15^N were quantified using a hierarchical model developed in a Bayesian framework. Jackson et al. (2011) pioneered multivariate ellipse‐based metrics to characterize isotopic niches (implemented in the *SIBER* library in R). Modeling isotopic standard ellipses in a Bayesian framework is considered to be particularly accurate when identifying isotopic niches at a colony level with a small sample size, i.e., with few sampled individuals (Jackson et al., 2011). In the present study, δ^13^C and δ^15^N stable isotope analyses were performed at the level of whisker segments, therefore providing intraindividual variability in isotopic composition over several months (cf. section concerning whisker growth above). Standard isotopic models (cf. Jackson et al., 2011) only consider interindividual variability to identify the isotopic niche at the species level, but we expanded this standard model to incorporate two levels of isotopic variability: an intraindividual level (characterized by several isotopic measurements along a whisker) and an interindividual one.

We assumed that isotopic data could be described by a bivariate normal distribution of mean μ and covariance matrix Σ (Jackson et al., 2011). In the formula, k denotes the *k* ^th^ species and i the *i* ^th^ individual; $n_{\mathrm{ik}}$ is therefore the number of isotopic measurements for individual i of species k. Also $\mu_{k}=\left\{\mu_{1k},\mu_{2k}\right\}$—the mean isotopic values of species k—with subscript 1 corresponding to carbon isotopic measurements, and 2 to nitrogen ones.

For each individual i of each species k,

(1) $$Y_{\mathrm{ik}}∼N_{2}\left(\alpha_{\mathrm{ik}},\Sigma_{k}\right)$$

where $N_{2}$ denotes a bivariate normal distribution of location parameters $\alpha_{\mathrm{ik}}$ and covariance matrix $\Sigma_{k}$. The correlation matrix $\Sigma_{k}$ allows for a residual‐level correlation between carbon and nitrogen isotopic measurements. Parameters $\alpha_{\mathrm{ik}}$ are individual‐specific mean isotopic values (so called “random effects”):

(2) $$\alpha_{\mathrm{ik}}∼S_{2}\left(n_{\mathrm{ik}},\mu_{k},\Omega_{k}\right)$$

where $S_{2}$ denotes a bivariate Student distribution of $n_{i}$ degrees of freedom, with location parameters $\mu_{k}$ and covariance matrix $\Omega_{k}$. The Student distribution allows for potential outliers (at the individual level): If there are few measurements for individual i, then the model allows for the possibility that this individual may be an outlier. The correlation matrix $\Omega_{k}$ allows for an individual‐level correlation between carbon and nitrogen isotopic measurements. Equations (1) and (2) define a hierarchical model that accounts for both intraindividual‐level correlation and interindividual‐level correlation via the covariance matrices $\Sigma_{k}$ and $\Omega_{k}$, respectively. For the latter, we used the prior of Huang and Wand (2013), to ensure a marginal uniform distribution on the correlation between carbon and nitrogen isotopic values:

$\Omega_{k}∼Wishart^{‐1}\left(3,\left[\begin{array}{cc} a_{1k} & 0\\ 0 & a_{2k} \end{array}\right]\right)\;\text{and}\;\Sigma_{k}∼Wishart^{‐1}\left(3,\left[\begin{array}{cc} b_{1k} & 0\\ 0 & b_{2k} \end{array}\right]\right).$

The priors for the variance parameters $\left\{a_{1k},a_{2k}\right\}$ and $\left\{b_{1k},b_{2k}\right\}$ were inverse gamma distributions $\Gamma^{‐1}\left(0.5,1.0\right)$ which induce a marginal half‐Student distribution with 2 degrees of freedom on the scale (i.e., the square root of a variance) parameters (Huang & Wand, 2013). Weakly informative priors were also used on location parameters $\mu_{1k},\mu_{2k}∼N\left(0.0,20.0\right)$. Parameter estimation was done using Hamilton Monte Carlo methods as implemented in software Stan (Carpenter et al., 2017). Four chains were initialized using the default options in package *rstan* (version 2.21.0, Stan Development Team, 2019) and run for a total of 2,000 iterations. The first 1,000 iterations served as warm‐up, and the remaining 1,000 were thinned to yield a sample of four draws per chain. Parameter convergence was assessed using the Gelman–Rubin–Brooks $\hat{r}$ statistics ($\hat{r}<1.05$).

### 2.5 Isotopic niche identification and interspecific overlap

Isotopic niches at the species level are operationalized as ellipses in δ^13^C and δ^15^N dimensions (with confidence interval of 95% in their drawing) and can be estimated from the joint posterior distribution of parameters {${\hat{\mu }}_{k},{\hat{\Omega }}_{k}$}. Variability at the individual level is summarized by the covariance matrix ${\hat{\Omega }}_{k}$ and should be included; otherwise, estimated isotopic niches will be too narrow if there are substantial differences in isotopic niches at the individual level within a given species. Isotopic values for 100 new individuals i were drawn from $\alpha_{\mathrm{ik}}^{\text{new}}∼N_{2}\left({\hat{\mu }}_{k}^{\left(j\right)},{\hat{\Omega }}_{k}^{\left(j\right)}\right)$, where j denotes the *j* ^th^ MCMC draw (iteration) from the joint posterior distribution; these values were then used to estimate isotopic niches. This procedure was repeated by drawing 1,000 iterations j from the posterior distribution to account for estimation uncertainty. We thus obtained a sample of 1,000 ellipses over which further inferences could be carried out. Interspecific overlap, in particular, and its associated uncertainty can easily be assessed, and any correlation between carbon and nitrogen isotopic values is automatically considered. We characterized the probability ranges of a point belonging to the isotopic niches (i.e., to the model ellipses) as well as the probability of interspecific niche overlap (each step is illustrated in Appendix 3).

The overlap between harbour seal and grey seal isotopic niches was quantified using the function “maxLikOverlap” in the package *SIBER* (version 2.1.5; Jackson et al., 2011) in R using the 1,000 ellipses generated for each species from the model. This function provides an estimate of the proportion of the whole isotopic niche area covered by both species that is overlapping, as well as the proportion of the first species' isotopic niche area in the second species' area, and vice versa.

### 2.6 Comparison of isotopic niches with isotopic values of potential prey

The isotopic values of some fish and cephalopod species, identified in the present study as preferred prey for harbour and grey seals (cf. diet analysis), were available for the EEC (Kopp et al., 2015). Kopp et al. (2015) measured the isotopic values of fish and cephalopods sampled at different depths in the EEC and presented results for different depth strata and for all depths pooled. We present the isotopic values of potential seal prey species for all depths except for *Clupea harengus* for which we present values for two sampled depth strata in which significant isotopic differences were identified (*C. harengus* in a benthic pathway at 0–20 m, and in a pelagic pathway at 20–38 m). We compared harbour and grey seal isotopic niches identified in this study with isotopic values of prey by applying a trophic enrichment factor (TEF), i.e., the increase in δ^13^C and δ^15^N values from prey to consumer. Assuming that TEFs vary depending on consumer species, prey, and analyzed tissues (Crawford et al., 2008), we used TEF values of +2.4 ± 1.3‰ for δ^13^C and +2.6 ± 1.2‰ for δ^15^N evaluated by Lerner et al. (2018) for grey seal whiskers using the SIDER method developed by Healy et al. (2018).

### 2.7 Identification of foraging areas

Tagging of seals was done after the molting period for each species—in October 2008 for harbour seals and in late May 2012 for grey seals (with the exception of two of the ten grey seals, G10 and G12, which were caught in September 2012) (Table 3). Tagging the seals just after molting allowed for the longest possible tracking duration, i.e., before the next molting period. At this stage, the seals' body reserves were also depleted following the molting season and they would therefore soon head out and increase their foraging activity to replenish their reserves, allowing us to identify their foraging areas and behaviors (Beck et al., 2003; Breed et al., 2009; McConnell et al., 1999; Thompson et al., 1994).

The eight harbour seals and ten grey seals were fitted with Fastloc™ GPS/GSM tags developed by Sea Mammal Research Unit (University of StAndrews, UK) to study their movements and foraging behavior. The tags provided data on their locations at the sea surface using a GPS sensor, as well as on their diving behavior. The following dive data were registered when a seal reached depths exceeding 1.5 m: start date and time, maximum depth, dive duration, the “percent area” (to calculate Time Allocation at Depth index, TAD; Fedak et al., 2001), and nine intermediate‐depth points (each 10% of the dive duration). Based on the analysis of this type of data, a “vertical approach” was developed to identify the likely foraging areas using some of the same individuals (Planque et al., 2020). Using two dive parameters—the TAD index describing dive shape and vertical descent speed—we selected the faster U‐shaped dives for each individual, as they likely represent foraging behavior. We defined likely foraging areas as the areas where the highest spatial densities of faster U‐shaped dives occurred (Planque et al., 2020). Individual foraging areas were identified throughout the total tracking period (average of 142 ± 53 days for harbour seal individuals and 143 ± 60 days for the grey seals; Table 3). Foraging areas for each individual are presented with kernel density contours at 95%, 75%, and 50% of the faster U‐shaped dives selected using the vertical approach. Note that the foraging areas of the grey seal G09 could not be identified due to a GPS malfunction on its tag.

## 3 RESULTS

### 3.1 Diet composition and interspecific overlap

**FIGURE 2:**
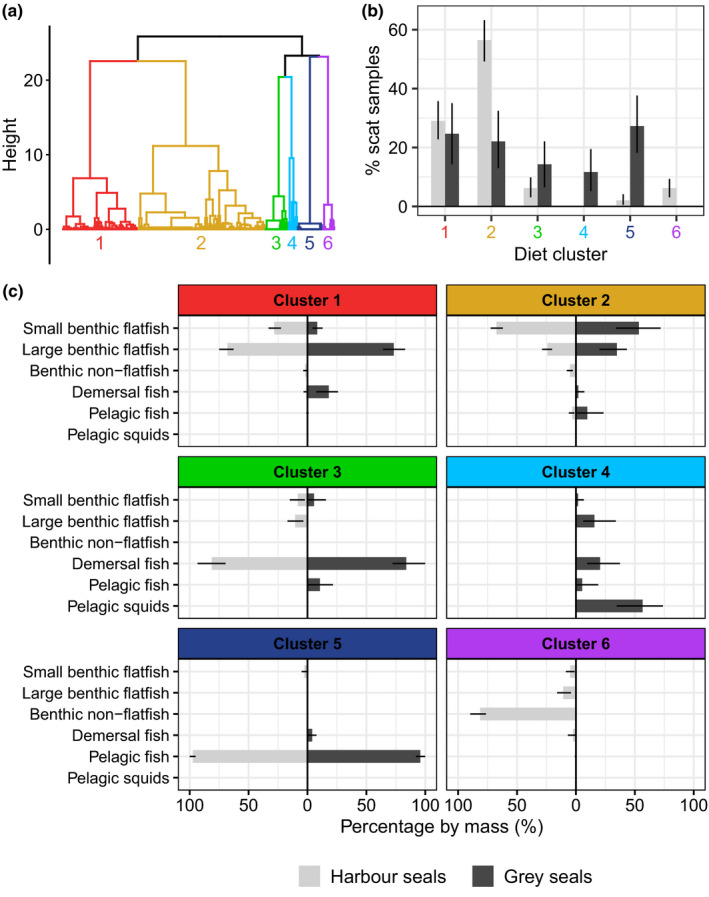
Dietary clusters identified from 193 harbour seal and 77 grey seal scat samples. (a) Identification of six diet clusters from scat samples according to prey composition (functional groups) by hierarchical clustering. (b) Percentage of scat samples in each diet cluster for both seal species with error bars indicating 95% confidence interval (CI95%). (c) The composition of each diet cluster, in percentage by mass, based on the scat samples associated with each cluster, error bars indicate 95% confidence intervals (CI95%)

Harbour and grey seal diet clusters (Figure 2a) are characterized by different patterns of prey functional group compositions, reflecting different typologies of scat content (Figure 2c). Scats from some clusters were almost exclusively composed of only one functional type of prey (clusters 3, 5, and 6), while others had more mixed content including different types of prey (clusters 1, 2, and 4). Descriptive details of harbour and grey seal scat contents at taxonomic prey species level are available in supplementary materials (Appendices 4 and 5).

Scats in clusters 1 and 2 mostly contained small and large benthic flatfish with a minority of other prey types (benthic non‐flatfish, demersal, and pelagic fish). The proportion (in mass) of large benthic flatfish (≥200 mm) was more significant than that of small flatfish (<200 mm) in scats of cluster 1 (~50%–75%), while small benthic flatfish were more significant than large flatfish in scats of cluster 2 (~50%–75%). Scats in cluster 3 almost exclusively included demersal fish and those in cluster 5 almost exclusively pelagic fish. Cluster 4 showed a high prevalence of pelagic squids, moderate quantities of demersal fish and large benthic flatfish, and some pelagic fish. Cluster 6 had a high prevalence of benthic non‐flatfish and small quantities of demersal fish and benthic flatfish.

Harbour and grey seal scats were unequally distributed in the diet clusters (Figure 2b). Most harbour seal scats (85.5% [CI95%: 80.3%–90.2%]) and around half of the grey seal scats (46.8% [35.1%–58.4%]) were associated with clusters 1 and 2, which are characterized by a high prevalence of small and large benthic flatfish. These two clusters were the only ones to which a high percentage of scats were associated for both seal species, i.e., the most interspecific dietary overlap occurs in clusters 1 and 2. Cluster 1, with a greater prevalence of large flatfish, accounted for around one quarter of harbour and grey seal scats (29.0% [22.8%–35.8%] and 24.7% [14.3%–35.1%] of scats, respectively). Cluster 2, however, with a greater prevalence of small flatfish, accounted for more than half of harbour seal scats (56.5% [49.2%–63.2%]), but only about a fifth of grey seal scats (22.1% [13.0%–32.5%]).

Clusters 3–6 were essentially characterized by a high proportion of other types of prey that are not benthic flatfish. These four clusters included half the grey seal scats (53.2% [41.6%–64.9%]) and only about one seventh of harbour seal scats (14.5% [9.8%–19.7%]). A substantial proportion of grey seal scats almost exclusively contained demersal fish (cluster 3; 14.3% [6.5%–22.1%]) and pelagic fish (cluster 5; 27.3% [18.2%–37.7%]), while very few harbour seal scats had this content pattern (6.2% [3.1%–9.8%] and 2.1% [0.5%–4.1%] for clusters 3 and 5, respectively). Only grey seal scats were included in cluster 4, with high pelagic squid content and moderate traces of fish (mostly demersal fish and flatfish), and these accounted for 11.7% [5.2%–19.5%] of all grey seal scats. Similarly, only harbour seal scats were included in cluster 6, with a high prevalence of benthic non‐flatfish and minor traces of other types of fish, which comprised only a small proportion of harbour seal samples (6.2% [3.1%–9.3%]).

We found a high degree of interspecific dietary overlap between harbour and grey seals based on the composition (in functional group) of their diet using the Pianka index (value when comparing all scat content data: 0.72 [0.53–0.82]).

**FIGURE 3:**
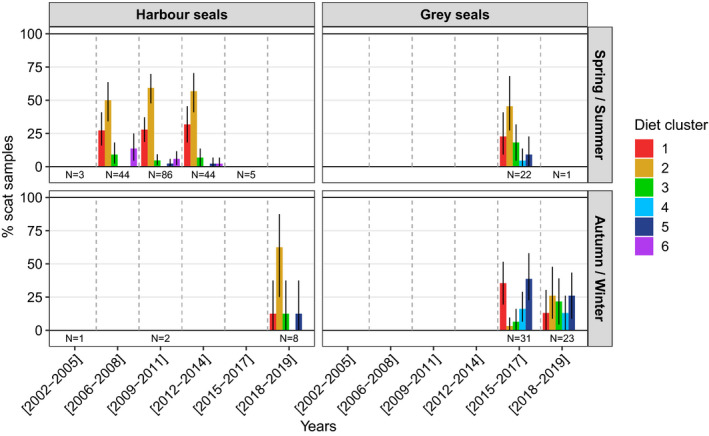
The percentage distribution of seal scat samples in each diet cluster along six 3‐year classes from 2002 to 2019, during spring/summer and autumn/winter periods, with CI95% error bars. The number of scats associated with each year‐class/season is indicated under the distribution bars. Only results based on at least 8 scats are presented here in order to reduce bias due to interindividual variations

Interseasonal and interannual variations in harbour and grey seal diets were depicted by plotting the scat distributions in each diet cluster by 3‐year classes, separately for spring/summer and autumn/winter (Figure 3). Only results based on at least eight scats per period were included here, although results with eight scats only provide a preliminary information on the diet (cf. harbour seal scats in autumn/winter of 2018–2019).

The proportions of harbour seal scats in each diet cluster in the spring/summer remained more or less stable over the three 3‐year periods between 2006 and 2014. High percentages of harbour seal scats contained significant traces of large and small benthic flatfish (clusters 1 and 2) over this time: 77.3% [65.9%–88.6%] in 2006–2008 (*N* = 44), 87.2% [79.1%–94.2%] in 2009–2011 (*N* = 86), and 88.6% [77.3%–97.7%] in 2012–2014 (*N* = 44). Diet cluster 2, marked by a greater prevalence of small benthic flatfish, dominated throughout the spring/summer of this period (from 50% [34.1%–63.6%] of scats in 2006–2008 to 59.3% [47.7%–69.8%] of scats in 2009–2011). Harbour seal diet therefore showed low interannual variability during the spring/summer period. Despite the small number of harbour seal scats collected in autumn/winter (*N* = 8 in 2018–2019) and the exclusion of distributions in 2002–2005 and 2009–2011 due to very low numbers of samples, we observed similarities with the pattern observed in spring/summer for this species as 75% [49.7%–100%] of these scats were part of clusters 1 and 2. The distribution of grey seal scats throughout the six diet clusters was more variable in autumn/winter than that of harbour seals in the spring/summer: a high percentage of scats collected in 2015–2017 were part of clusters 1 (35.5% [19.4%–51.6%]) and 5 (38.7% [22.6%–58.1%])—containing significant traces of large benthic flatfish and pelagic squids, respectively—while scats from 2018 to 2019 were relatively evenly distributed among the diet clusters (13% [0%–30.4%] for cluster 1, 26.1% [8.7%–47.8%] for cluster 2, 21.7% [4.3%–39.1%] for cluster 3, 13% [0%–26.1%] for cluster 4, and 26.1% [8.7%–43.5%] for cluster 5). Of the grey seal scat samples, collected between 2015 and 2019, only 29.9% were found in the spring/summer, of which only one was found in 2018–2019. The distribution pattern we see during the spring/summer of 2015–2017 did not match the patterns in scats of either distribution in autumn/winter as high proportion of these scats contained significant traces of large and small benthic flatfish (22.7% [9.1%–40.9%] for cluster 1 and 45.5% [27.3%–68.2%] for cluster 2). Grey seal scats therefore showed higher interannual and interseasonal variability in percentages of scats distributed throughout the diet clusters than harbour seal scats.

### 3.2 Characteristics of captured individuals

All seals sampled for stable isotope and foraging area analyses aside from one harbour seal were male (Table 3). The average harbour seal body mass was 82 ± 12 kg, and average body length was 141 ± 6 cm with little interindividual variation, suggesting all individuals were adults or young adults. Grey seals showed higher interindividual differences in body mass and length: the average mass and length of five of the ten individuals (G01, G03, G05, G09, and G10) was 85 ± 21 kg and 150 ± 8 cm, respectively, while the other five (G02, G04, G07, G08, and G12) weighed 163 ± 26 kg and measured 190 ± 13 cm on average. These latter individuals are likely adults based on the relationship between body size and age (Murie & Lavigne, 1992).

### 3.3 Isotopic niche overlap

**FIGURE 4:**
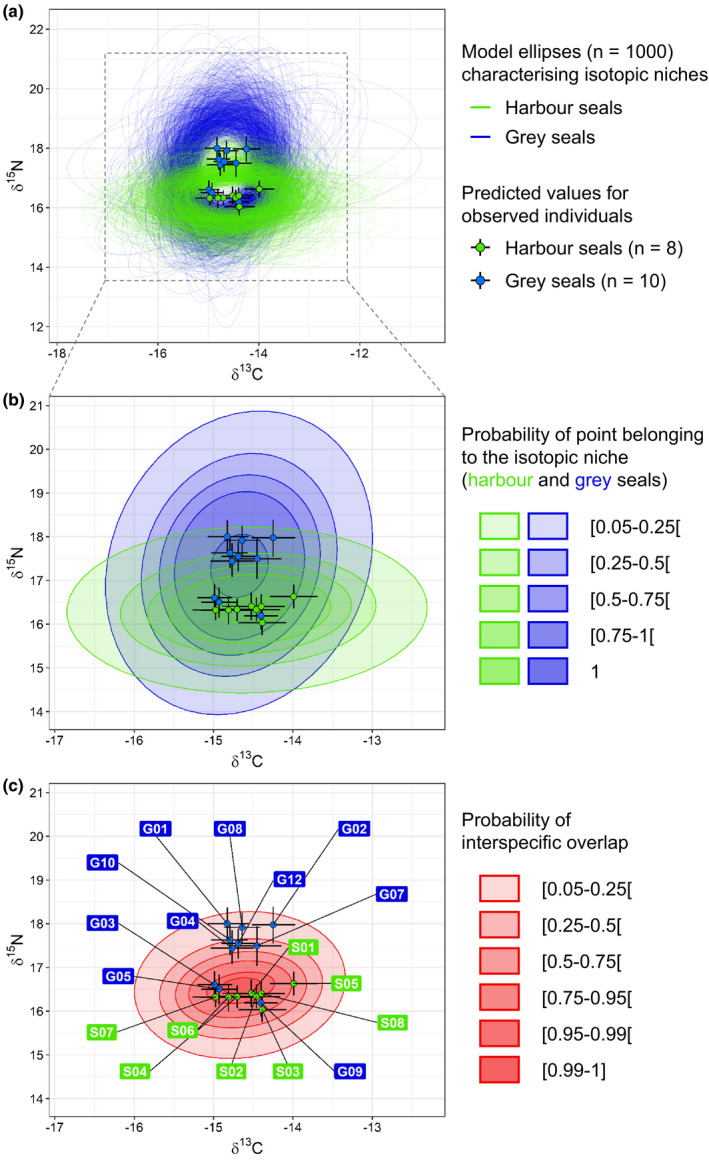
Isotopic niches of harbour and grey seals estimated using Bayesian modelling run on δ^13^C and δ^15^N stable isotope ratios measured along a whisker of eight harbour seal and ten grey seal individuals. (a) Isotopic niches characterized by standard ellipses at a 95% confidence interval for harbour seals (green) and grey seals (blue). (b) Ranges of probability for isotopic niches of harbour and grey seals. (c) Ranges of probability of interspecific isotopic niche overlap. Each probability range in b and c was characterized by ellipses at 95% around uniform points describing this probability (see Appendix S3). Points in a, b, and c are averages of predicted isotopic values for observed harbour and grey seal individuals, and confidence intervals are given at 95%

Harbour and grey seal isotopic niches differed in width (Figure 4a). The isotopic niche of harbour seals was characterized by an area of 3.88‰^2^ [CI95%: 1.09–8.17‰^2^] and that of grey seals by an area of 5.93‰^2^ [2.32–10.82‰^2^]. The probability of the grey seals' isotopic niche being larger than that of harbour seals was 0.78. Of the total niche area, 26.6% [8.8%–45.3%] was overlapping. A larger area of the harbour seal isotopic niche was nested within the grey seal niche (58.2% [22.7%–100%]) compared with the area of the grey seal isotopic niche nested within the harbour seal niche (36.3% [11.1%–63.5%]). The probability of the harbour seal isotopic niche being more nested within the grey seal niche than the reverse was 0.78.

Figure 4b shows the probabilities of a point belonging to harbour and grey seals' isotopic niches. By calculating the probabilities of a point belonging to both isotopic niches, we identified the probability of these niches overlapping (Figure 4c).

Interindividual differences in δ^13^C in isotopic niches were characterized by the scale parameters (in matrix Ω) of 0.54‰ [0.24–0.94‰] for harbour seals and 0.41‰ [0.21–0.66‰] for grey seals, with a probability of 0.72 that differences would be higher for harbour seals. A higher interindividual variability in δ^15^N within the isotopic niche was noted for grey seals (0.83‰ [0.44–1.31‰]) than for harbour seals (0.41‰ [0.18–0.74‰]). The probability of interindividual variability in δ^15^N being higher in the grey seal isotopic niche than the harbour seal niche was 0.94. Most of the isotopic niche divergence observed between the two species resulted from the larger size of grey seal isotopic niche which was more extensive in δ^15^N (Figure 4b).

Almost all of the harbour seal individuals in this study had predicted isotopic values within the core of the highest probability of interspecific overlap (Figure 4c). Three of the ten grey seals (G03, G05, and G09) had predicted isotopic values close to harbour seal values, thus also within the range where the probability of interspecific overlap was high. The other seven grey seals were characterized by higher predicted δ^15^N values and were therefore in the range where the probability of interspecific overlap was lower. Seal isotopic niches were additionally identified by excluding these three grey seals G03, G05, and G09 from the input data (and keeping all harbour seal data), and this test showed a substantial decrease in isotopic overlap in comparison with the initial case with all grey seal data (cf. Appendix 6). The overlap therefore decreased to 11.1% [0%–28.2%] of the total niche area when excluding these individuals (vs. 26.6% [8.8%–45.3%] with all grey seals), and the probability of having a lower overlap was 0.88. The proportion of harbour seal niche area nested within the grey seal niche decreased to 23.1% [0%–68.1%] when excluding the three grey seals (vs. 58.2% [22.7%–100%] with all grey seals), and the probability of having a lower overlap was 0.86. The proportion of grey seal niche area nested within the harbour seal niche also decreased to 20.1% [0%–52.7%] in that case (vs. 36.3% [11.1%–63.5%] with all grey seals), and the probability of having a lower overlap was 0.79.

### 3.4 Comparison of isotopic niches with isotopic values of potential prey

**FIGURE 5:**
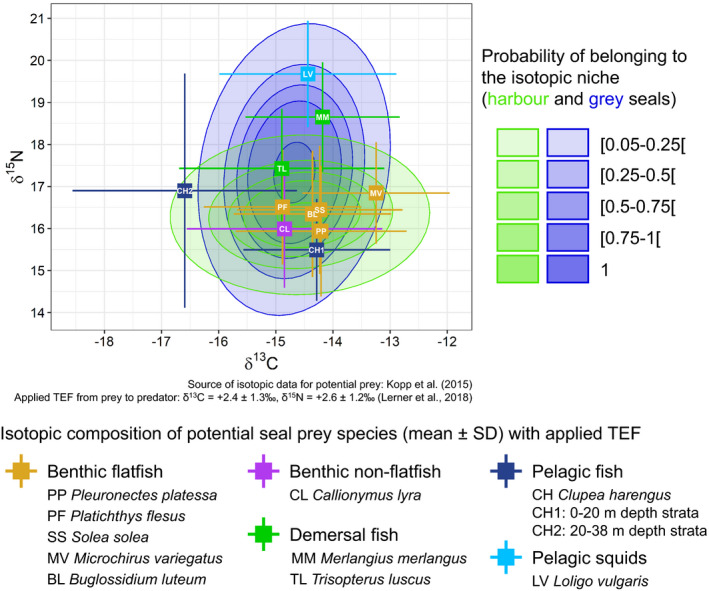
Comparison of harbour and grey seal isotopic niches (see Figure 4b) with isotopic values of potential prey in the Eastern English Channel. Isotopic data of potential prey from Kopp et al. (2015). TEF, Trophic Enrichment Factor

The isotopic compositions of potential seal prey species, after application of trophic enrichment factors (TEFs), were located within the probability ranges of harbour and grey seal isotopic niches (Figure 5). The benthic flatfish species, which constituted a large part of harbour seals' diet (see Section 3.1; Figure 2), had isotopic values (+TEF) in the higher probability ranges of harbour seals' isotopic niche, except for *Microchirus variegatus* which were in the lower probability ranges. These species were also identified as part of the grey seals' diet, and their isoptic values (+TEF) were also within the high probability ranges of the grey seals' isotopic niche. Two demersal fish species had isotopic values (+TEF) in the higher probability ranges of the grey seals' isotopic niche; however, they were either in the lower probability range (*Trisopterus luscus*) or entirely outside (*Merlangius merlangus*) of the harbour seals' isotopic niche. The pelagic squid *Loligo vulgaris*, identified as a potential prey for grey seals (Figure 2), had isotopic values (+TEF) exclusively within the grey seals' isotopic niche. Benthic non‐flatfish *Callionymus lyra* occurred only in harbour seals' diet (Figure 2) and was therefore considered as a potential prey only for harbour seals; however, the isotopic value of this species was within the isotopic niche of both species. Similarly, while the pelagic fish *C. harengus* was almost exclusively identified in grey seal diet (Figure 2), its isotopic value based on individuals found in shallower strata (0–20 m, detected in a benthic pathway; cf. Kopp et al., 2015) was within both seal species' niches. However, the isotopic value of *C. harengus* from deeper strata (20–38 m, detected in a pelagic pathway) was right on the outer edges of harbour and grey seal isotopic niches.

### 3.5 Foraging areas

**FIGURE 6:**
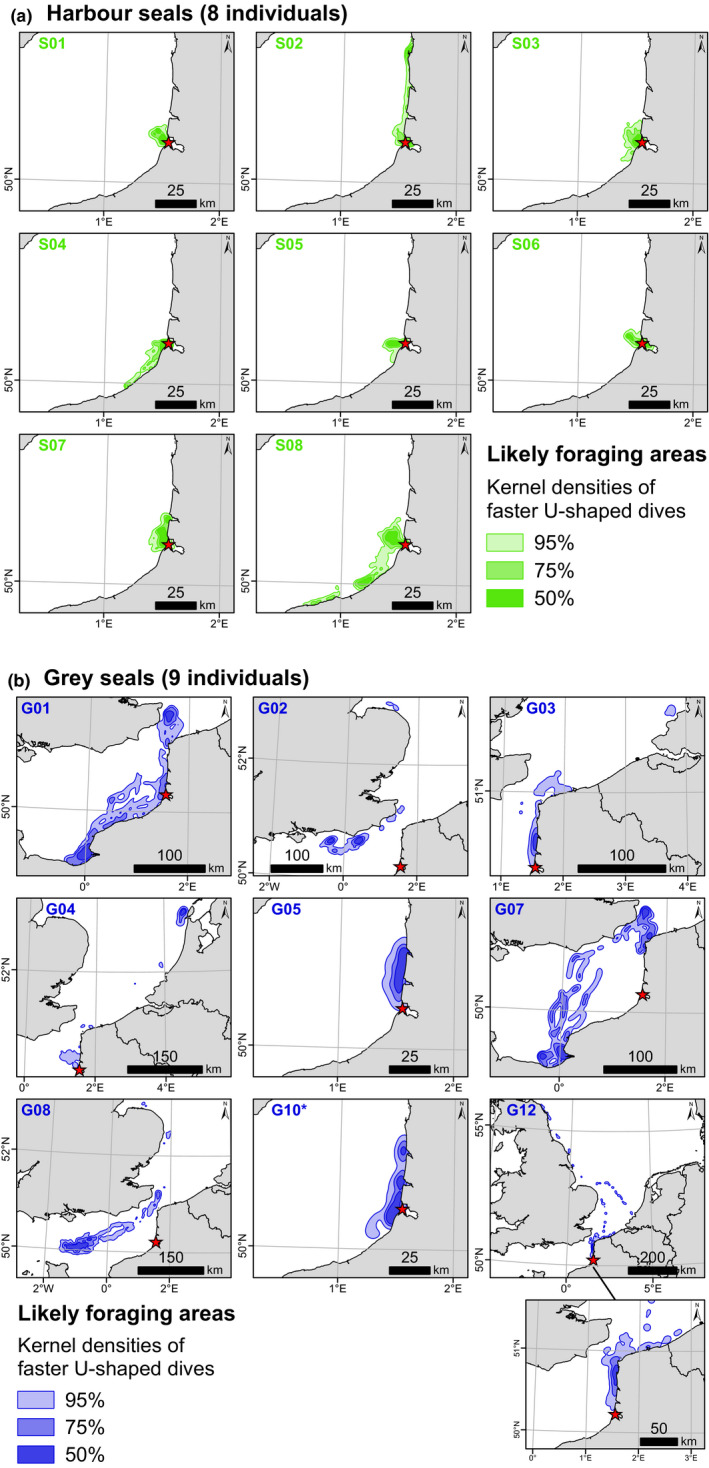
Foraging areas of eight harbour seals (a) and nine grey seals (b) captured in the Baie de Somme in 2008 and 2012, respectively, and tracked with GPS/GSM tags (Sea Mammal Research Unit, University of St Andrews, UK). Likely foraging areas of each individual are characterized by spatial kernel densities (50%, 75%, 95%) of faster U‐shaped dives. *Grey seal G10 was tracked for only 24 days, so its foraging areas during that time may not fully characterize this individual's foraging habits. Red stars show the location of the Baie de Somme, the capture site

Harbour seals foraged mostly in the coastal area near the Baie de Somme (BDS) during the tracking period (142 ± 53 days on average) (Figure 6a). Three of the eight harbour seals (S02, S04, and S08) ventured a bit further away from the capture site to forage, remaining close to the shore, but the majority of foraging occurred close to their site of capture.

Grey seal foraging areas were characterized by high interindividual differences in location and extent (Figure 6b). Grey seals were tracked for an average of 143 ± 60 days, and grey seal G10, however, was only tracked for 24 days. Five of the grey seals foraged in more extensive areas than harbour seals, either within a larger area including the BDS coastal area (G01 and G04) or only within areas over a hundred kilometers from the BDS (G02, G07, and G08). The four other grey seals (G03, G05, G10, and G12) foraged at a smaller spatial extent, in coastal areas close to the BDS and the two estuaries to the north (Baie d'Authie and Baie de Canche; see Figure 1). Their foraging areas were therefore more similar to harbour seal foraging areas, partially overlapping with this species. It is notable that grey seals G03 and G05 were two of the three individuals for which predicted δ^13^C and δ^15^N values were similar to harbour seals, i.e., associated with high probability of interspecific overlap (Figure 4c). The third individual was G09, the one for which we could not identify foraging areas due to a tag malfunction. Among the coastal grey seals, note that G12 also showed less dense and smaller foraging areas in the North Sea, while 75% of its likely foraging dives remained coastal and close to the capture site.

## 4 DISCUSSION

This study reveals a high trophic niche overlap between sympatric harbour and grey seals at the southern limit of their European range. Results obtained from the three complementary approaches (based on diet composition, isotopic niches, and foraging areas) were in agreement and suggested that the larger niche of grey seals covered most of the harbour seal niche, which was narrower. This overlap was associated with a specific foraging strategy—feeding on benthic flatfish in coastal waters—and we suggest that this key finding provides the context for potential competition, either current or future, between the two species. Continued increase in the number of grey and harbour seals and/or a decrease in flatfish supply in this area could imply/amplify competitive interactions and impact populations.

### 4.1 Trophic niche overlap identified by different approaches

The three approaches used in this study provided converging results on the trophic niche overlap between sympatric harbour and grey seals at the southern limit of their European range. The cause of this overlap was identified to be the consumption of benthic flatfish by both species. Scat content analysis provided an initial quantification of the overlap in diet and highlighted the type of prey likely causing the overlap: benthic flatfish. The identification of harbour and grey seal isotopic niches provided another way to quantify and evaluate the strength of the overlap in diet. Comparing seal isotopic niches with the isotopic values of potential prey species—detected in scat samples—further supports that it is the consumption of benthic flatfish which gives rise to the trophic niche overlap between harbour and grey seals. The overlap in isotopic niches could result from consumption of prey species with isotopic values similar to benthic flatfish, detected almost exclusively in harbour seal (*C. lyra*, benthic non‐flatfish) or in grey seal (*C. harengus*, pelagic fish, on 0–20 m depth strata) diets. These findings may also provide more detailed insights into the foraging ecology of seals. For example, Kopp et al. (2015) found strong isotopic differences between *C. harengus* in shallower waters (0–20 m) where they feed in the benthic pathway and those in deeper waters (20–38 m) foraging in the pelagic pathway. Taking this and the isotopic niches into account, it is likely that grey seals caught this herring species in shallower waters (see Figure 5). Results from the various approaches therefore have to be interpreted in synergy, using diet composition data to provide information that could not be elucidated from isotopic niches alone and vice versa. The complementarity between diet composition and isotopic niches in this study illustrates the importance of using multiple approaches focusing on various metrics to accurately characterize trophic niche overlap (e.g., Costa‐Pereira et al., 2019).

Each approach used in this study is based on data differing in nature and structure and thus cannot provide a robust prediction of overlap individually as different spatial and temporal scales are involved, as well as variations in sample sizes and sampling periods. The challenge in using these different methodologies was therefore to characterize trophic niches at the seal colony/population level with the highest possible confidence, despite the limits of the methodologies and data.

Each seal scat provides a snapshot of dietary events that occurred several hours to several days before sampling. The time elapsed from the time of consumption could not be defined as the residence of prey remains may vary depending on prey type and/or part type (Harvey, 1989). Linking a precise time and location to these dietary events is almost impossible. While a single scat characterizes dietary events at low spatial and temporal resolutions, the addition of several samples collected at different times provides a more extensive temporal view of foraging patterns similar to isotopic niches, but for more individuals than isotopic analyses using whiskers. Yet, we have to assume that different scats could potentially have come from the same individual, and thus, the content of each scat does not uniquely characterize an individual's foraging strategy but rather provides a snapshot of what one individual ate at a certain time. Since diet results can be strongly influenced by the number of seal scats collected, we acknowledge that the number of samples collected in some seasons was low and was unbalanced (Figure 3). Difficult field conditions (including low number of seals) and regulations around disturbing seals too frequently (both seal species are protected at a national and at Community level) in protected areas limited sampling opportunities and undermined the possibility to have a balanced sampling across seasons and species. Regardless of this, we still highlighted a strong stability in harbour seal diet in spring/summer between 2006 and 2014—when the number of harbour seals more than doubled and when grey seals increasingly started arriving at the study site (Vincent et al., 2017). The dietary observations over that time provided strong evidence that benthic flatfish (particularly small ones from nurseries) are of major importance for this species during this season (Spitz et al., 2015). The more recent efforts to study grey seal diets through the seasons revealed that benthic flatfish also constituted a high proportion of their diet, substantiating the dietary overlap with harbour seals. We tried to create a more balanced view of harbour seals' diet by increasing sampling of scats during autumn/winter toward the end of the study period, and these preliminary results showed that their autumn/winter diet might be similar to their spring/summer diet (mostly including benthic flatfish) but we recognize that the number of scats (*N* = 8) was too low to conclude this with reasonable certainty. We are therefore cautious in interpreting seasonal variations in the diets of both species.

The low numbers of whiskers collected for isotopic niche determination led us to work in a Bayesian framework, considering interindividual variability (Jackson et al., 2011). Working in a probabilistic framework provided more accuracy in the identification of seal isotopic niches and overlap. We incorporated a medium temporal view of the trophic niche, a novel aspect of isotopic niches, by including the intraindividual variability in foraging patterns in the model (time‐integrated measures of isotopes, cf. information on whisker growth in Section 2.4). Isotopic niches incorporated a wide spatial dimension by integrating several months of whisker growth, i.e., several months of foraging. However, these niches were determined using whiskers collected from seal individuals captured for telemetry studies, and thus, sampling was driven by the constraints and objectives of such studies (attachment of the tags to the fur just after their molt; see Section 2.7). Harbour and grey seal isotopic niches therefore would not characterize the same time integration (lower for harbour seals) and period, and the harbour seal niche could represent the foraging patterns of the individual up to around 4 months prior to the time capture (i.e., during summer/early autumn 2008) while the grey seal niche around 1 year before capture (i.e., from late spring 2011 to spring 2012). However, there are high uncertainties in the growth patterns of both species whiskers due to a lack of knowledge, including that some seal whiskers might be characterized by a biennial retention and could therefore have grown during a longer period than what we estimated; thus, the time integration of isotopic niches has to be interpreted carefully. Still, we are confident that the isotopic niches of both harbour and grey seals would include the summer, when there is believed to be higher trophic overlap between the two species as there are more grey seals in the Eastern English Channel (EEC) during this season, while harbour seals are present year‐round (Vincent et al., 2017). Another possible limitation is that isotopic values of prey consumed could vary interannually (e.g., Kurle et al., 2011), which could have impacted harbour and grey seal isotopic niches in 2008 and 2011–2012, respectively; however, to our knowledge, there are no data of any such shifts in the EEC and we think that significant variations are unlikely given the high concordance observed between results from all approaches used.

The foraging areas identified for the individuals we sampled showed an overlap in feeding grounds in the coastal waters around the Baie de Somme (BDS) haulout site, where all eight harbour seals focused their foraging effort, and four of the nine grey seals spent considerable time (G03, G05, G10, and G12) as part of their more extensive feeding areas. This is consistent with the general spatial foraging patterns observed elsewhere for these species (e.g., Cunningham et al., 2009; Härkönen & Harding, 2001; Thompson et al., 1996). The results of the foraging area and diet analyses support the findings of the isotopic niche analysis. The narrow isotopic niche of harbour seals nested within the larger niche of grey seals is in line with the diet and foraging patterns we observed, namely that harbour seals ate relatively unvaried diets, including high percentages of benthic flatfish (especially small ones), and they foraged within closer ranges to the haul out site (where flatfish nurseries are located; e.g., Riou et al., 2001; Rochette et al., 2010), while grey seals had more diverse foraging strategies with some individuals foraging a fair amount close to the BDS, and their diets included more diverse prey types but still a notable portion of benthic flatfish (that can be both in nurseries in coastal waters, or in deeper offshore waters when recruited to the adult stage; e.g., Riou et al., 2001; Rochette et al., 2010). The similarity in isotopic values and foraging areas of grey seals G03 and G05 to those of harbour seals (Figure 4c and Figure 6) further supports the understanding that the isotopic niche overlap is due to feeding on similar prey (of similar isotopic values) in a similar area. Thus, the broader trophic niche for grey seals and the larger extent of their foraging areas, as well as the overlap that could arise from specific individuals, are in line with specialist feeding behaviors observed in grey seals at the individual level (Gosch et al., 2014; Hernandez et al., 2019; Tucker et al., 2007).

These inferences, however, are based on a low number of individuals so we remain cautious in our interpretations. Results from more individuals are needed to confirm our findings with more confidence. We are also aware of potential limitations due to foraging areas and isotopic niches not being studied over the same time period (isotopic niches before capture and whisker sampling, and foraging areas afterward, following tagging) nor over the same season, but the benefit of this sampling structure is that we could track grey seals in summer when interspecific overlap is likely greatest in this area. We assumed in this study that foraging areas identified for grey seals reflected the particular foraging habits of individuals and that patterns observed would be similar to those of the previous spring/summer (the period for which we determined isotopic niches). We also assumed that the restricted foraging areas of harbour seals (shown here for autumn/winter/spring) would have been similar or more restricted within the area close to the haulout site in summer as they would have been breeding and molting (Thompson et al., 1994; Van Parijs et al., 1997).

The age and sex of individuals could also have skewed isotopic niche and foraging area results at the colony/population scale, especially for harbour seals which included almost exclusively adult males (seven out of eight individuals). There are no sex ratio data for harbour seals in the BDS, but since this colony is sedentary and reproductive we know that adult females are present year‐round, remaining close to the haulout sites during breeding especially (late spring/early summer), and we acknowledge that female foraging behavior could be underrepresented in isotopic niches and foraging areas. However, it is likely that trophic niches of male and female harbour seals are similar. Based on scat samples from the BDS, Spitz et al. (2015) found that males and females (sex assignment by molecular method) had similar overall diet compositions by mass despite a higher diversity of secondary prey species (species other than flatfish) in male scats. All grey seals tagged and sampled were males, which accurately reflects the biased sex ratio of the subpopulation in the EEC (high majority of adult males; Vincent et al., 2018); therefore, the grey seal isotopic niche may be a more accurate representation of the colony/population. There was also a size disparity among grey seals sampled (from 140 to 205 cm), and the three individuals (G03, G05, and G09) that had predicted isotopic values close to harbour seal values were among the smallest (140–162 cm) and lightest (61–112 kg) individuals. A possible hypothesis could therefore be that the trophic niche overlap between both seal species might be due to young grey seals. The number of individuals captured is too low, however, to fully test or confirm this bias due to age classes.

### 4.2 Ecological implications

In identifying and quantifying the trophic niche overlap between sympatric harbour and grey seals (here at their southern range limit), we aimed to highlight any trophic similarities or differences within a context where the two species are potential competitors. If circumstances change such as to create or increase the competition between these two species, grey seals may have the advantage over harbour seals in this case, as already suggested (e.g., Svensson, 2012; Wilson & Hammond, 2019). The wide overlap in harbour and grey seal diets we found, likely driven by the significant consumption of benthic flatfish by both species, indicates that competitive interactions around this type of prey cannot be excluded. The approach used in this study therefore provides a useful tool with which to identify the potential for interspecific competition (i.e., identifying shared prey types for which species could compete, and where competition is most likely) but it does not provide explicit proof of competition.

The findings of this study stand in contrast to what we have seen in their core distribution in the North Sea, where local harbour seal declines were observed as early as the 2000s. Declines in sandeel abundance were presented as a potential cause of these local harbour seal declines in the North Sea, sandeels having been a significant part of both species' diet prior to these declines (Wilson & Hammond, 2019). Recent findings have suggested that there is trophic segregation between the species in the Scottish and German parts of the North Sea, with grey seals continuing to feed on sandeels in offshore waters, while harbour seals forage more inshore on a wider range of prey types (Damseaux et al., 2021; Wilson & Hammond, 2019). Assuming that there is competition between the two species, differences between the patterns of overlap observed in the seals' core European distribution and at their southern range limit could be due to differences in the stage of the population: established colonies at the core, where effects of interspecific competition have potentially already been observed (decline in harbour seals) versus more recent arrival of seals at the range limit where competition may be occurring but measurable effects in population dynamics have not yet manifested.

This study was the first assessment of harbour and grey seal trophic niches and their overlap during a period of exponential increase in both species in the BDS, with continuing establishment of a reproductive harbour seal colony and the concomitant arrival of visitor grey seals from the North Sea (Vincent et al., 2017). As such, we report the seals' foraging ecology prior to potential modifications in their populations in the EEC resulting from various causes, including changes in resource availability and trophic interactions.

We suggest that flatfish stocks may currently be sufficient to maintain both species and potentially continue maintaining growing population numbers in the EEC. However, the EEC is subject to strong and quick ecological shifts, likely due to anthropogenic causes (e.g., climate change; Auber et al., 2017; McLean et al., 2019). Drastic declines in fish abundance have been observed these last decades in the BDS (decline by 80% for the last 30 years) as well as major changes in the functional organization of fish nurseries (McLean et al., 2019). Benthic flatfish in the EEC could therefore become a limited resource for harbour and grey seals in the near future if grey seal numbers continue to increase exponentially and they focus their foraging effort on this prey type, and/or if flatfish stocks continue to decrease. Too much pressure on flatfish stocks in the BDS could impact harbour seals in the same way that the decline of the sandeel is believed to have had in the North Sea population (Wilson & Hammond, 2019).

Competition between two species could induce the exclusion of the one that does not succeed to adapt (Gause, 1932), suspected to be harbour seals in this duo. Harbour seals in the BDS focused most of their foraging effort on benthic flatfish (especially from nurseries in coastal waters), which potentially reflects the foraging strategy at the colony scale (Spitz et al., 2015). How vulnerable they are to potential competition if benthic flatfish decline in coastal estuaries depends on their ability to adapt. Harbour seals are considered generalist feeders (Kavanagh et al., 2010; Olsen & Bjørge, 1995), it is thus possible that harbour seals could adapt to resource scarcity by foraging on other prey available in the EEC (demersal and pelagic fish, pelagic squids). However, harbour seals would then be foraging on trophic dimensions (e.g., prey species, foraging areas) already and increasingly occupied by grey seals, which could impact harbour seal foraging success and possibly their population dynamics. Ecological shifts would lead to changes in grey seal trophic niches when seals compensate for the lack of benthic flatfish, therefore simultaneously decreasing availability of prey within the niche of both seal species and potentially increasing interactions. Being at their range limit, these species should by definition be faced with conditions that are limiting, including a lower density of resources compared to at their core distribution. If there is competition currently between harbour and grey seals around the BDS, we can expect to see visible effects of this in the future, including shifts in diet, foraging areas, and/or population dynamics of harbour seals. Monitoring harbour and grey seal trophic niches, foraging areas, and population trends in the coming years will be essential to identify potential changes resulting from competition for prey.

## CONFLICT OF INTEREST

The authors declare that they have no conflict of interest.

## AUTHOR CONTRIBUTIONS

**Yann Planque:** Conceptualization (lead); Data curation (equal); Formal analysis (lead); Investigation (lead); Methodology (lead); Validation (equal); Writing‐original draft (lead); Writing‐review & editing (lead). **Jérôme Spitz:** Conceptualization (supporting); Data curation (lead); Formal analysis (supporting); Funding acquisition (equal); Investigation (equal); Methodology (equal); Validation (equal); Writing‐original draft (equal); Writing‐review & editing (equal). **Matthieu Authier:** Conceptualization (supporting); Formal analysis (equal); Investigation (supporting); Methodology (equal); Validation (equal); Writing‐original draft (equal); Writing‐review & editing (equal). **Gaël Guillou:** Data curation (equal); Investigation (supporting); Validation (supporting); Writing‐review & editing (equal). **Cécile Vincent:** Conceptualization (supporting); Data curation (lead); Formal analysis (supporting); Funding acquisition (lead); Investigation (equal); Methodology (supporting); Project administration (lead); Supervision (lead); Validation (equal); Writing‐original draft (equal); Writing‐review & editing (equal). **Florence Caurant:** Conceptualization (supporting); Data curation (lead); Formal analysis (supporting); Funding acquisition (lead); Investigation (equal); Methodology (supporting); Project administration (lead); Supervision (lead); Validation (equal); Writing‐original draft (equal); Writing‐review & editing (equal).

## ETHICAL APPROVAL

All procedures involving harbour and grey seals were in accordance with the ethical standards of the French Ministry of the Environment, and seals were caught and instrumented under license numbers 08/346/DEROG and 11/874/DEROG, delivered by this Ministry.

### OPEN RESEARCH BADGES

This article has been awarded Open Data and Open Materials Badges. All materials and data are publicly accessible via the Open Science Framework at diet data from https://doi.org/10.17882/76780, stable isotopes data from https://doi.org/10.17882/76528, dive data from https://doi.org/10.17882/80016 and diet analysis from https://github.com/YannPlanque/Diet_Cluster_and_Overlap, isotopic niche analysis from https://github.com/YannPlanque/Isotopic_Niche_Overlap, and dive data analysis to identify individual foraging areas (based on the methodology by Planque et al., 2020) from https://github.com/YannPlanque/Foraging_Areas_with_Dive_Data (written in R language).

## Supporting information

Appendix S1‐S6

## Data Availability

The data used in this study are permanently available on SEANOE repositories under the license “Creative Commons BY‐NC” and are freely downloadable: diet data from https://doi.org/10.17882/76780, stable isotopes data from https://doi.org/10.17882/76528, and dive data from https://doi.org/10.17882/80016. The three scripts developed in this study to perform all presented analyses are freely available on GitHub repositories: diet analysis from https://github.com/YannPlanque/Diet_Cluster_and_Overlap, isotopic niche analysis from https://github.com/YannPlanque/Isotopic_Niche_Overlap, and dive data analysis to identify individual foraging areas (based on the methodology by Planque et al., 2020) from https://github.com/YannPlanque/Foraging_Areas_with_Dive_Data (written in R language).
