# Supplementary material for: Trophic niche overlap between sympatric harbour seals (Phoca vitulina) and grey seals (Halichoerus grypus) at the southern limit of their European range (Eastern English Channel)
Source: Ecol Evol. 2021 Jul 5;11(15):10004–25. doi: 10.1002/ece3.7739 (PMC8328439; doi:10.1002/ece3.7739)
Supplement: Supplementary file 1 — Appendix S1‐S6 [file ECE3-11-10004-s001.pdf]

– Ecology and Evolution –

Trophic niche overlap between sympatric harbour seals (*Phoca vitulina*) and grey seals (*Halichoerus grypus*) at the southern limit of their European range (Eastern English Channel)

<u>Short title</u>: Trophic niche overlap between two seal species

Yann Planque[1*], Jérôme Spitz[1,2], Matthieu Authier[2,3], Gaël Guillou[4], Cécile Vincent[1], Florence Caurant[1,2]

[1] Centre d'Études Biologiques de Chizé, CEBC, UMR 7372 CNRS / La Rochelle Université, 5 allée de l'Océan, 17000 La Rochelle, France

[2] Observatoire Pelagis, UMS 3462 CNRS / La Rochelle Université, 5 allée de l'Océan, 17000 La Rochelle, France

[3] ADERA, 162 Avenue du Dr Albert Schweitzer, CS 60040, 33608 Pessac Cedex, France

[4] Littoral Environnement et Sociétés, LIENSs, UMR 7266 CNRS / La Rochelle Université, Avenue Michel Crépeau, 17000 La Rochelle, France

*Contact: yann.planque@univ-lr.fr; yann.planque@hotmail.fr

Supplementary materials

Appendix 1 Allometric relationships used to reconstruct body mass (FM) and body length (FL) of prey ingested by harbour and grey seals from measurements of fish otoliths (otolith length OL or otolith width OW) and cephalopod beaks (lower rostral length LRL) in mm. The source of each allometric relationship is mentioned.

Functional group	Family	Prey	Type of diagnostic hard part measured<br>OL: Otolith Length<br>OW: Otolith Width<br>LRL: Lower Rostral Length<br>(in mm)	Allometric relationships between OL or OW or LRL with body mass (FM) and body length (FL)<br>Fish/Cephalopod body mass (FM) (in g)	Fish/Cephalopod body length (FL) (in mm)	Source
Small benthic flatfish (relative length < 200 mm)<br>or<br>Large benthic flatfish (relative length ≥ 200 mm)	Pleuronectidea	*Pleuronectes platessa* / *Platichthys flesus*	OL	FM = 0.498 * (OL^3.408)	FL = -3.81 + 47.63 * OL	Härkonen (1986)
	Soleidae	*Solea solea* / *Pegusa lascaris*<br>*Buglossidium luteum* / *Microchirus variegatus*	OL	FM = 2.535 * (OL^3.444)	FL = -12.622 + 80.901 * OL	Härkonen (1986)
	Bothidea	Und. Bothidea<br>*Scophtalmus* spp.<br>*Phrynorhombus norvegicus*	OL	FM = 1.4 * (OL^3.2)	FL = -11.42 + 54.77*OL	Härkonen (1986)
Benthic non-flatfish	Gobidae	Und. Gobidae	OL	FM = 0.1677 * (OL^5.369)	FL = -13 + 50 * OL	Observatoire Pelagis
	Trachinidae	*Trachinus draco*	OL	FM = 2.0558 * (OL^2.0631)	FL = (2.1186 * OL + 6.061) * 10	Observatoire Pelagis
	Callionymidae	*Callionymus lyra*	OL	FM = 0.482 * (OL^4.459)	FL = 44.29*(OL^1.412)	Härkonen (1986)
	Triglidae	Und. Triglidae	OL	FM = 1.007 * (OL^3.616)	FL = (2.1186 * OL + 6.061) * 10	FM: Härkonen (1986)<br>FL: Observatoire Pelagis
Demersal fish	Gadidae	*Merlangius merlangus*	OW	FM = (0.88 * OW)^3.73	FL = (-2.97 + 6.74 * OW) * 10	Coull et al. (1989)
		Trisopterus spp.	OL	FM = 0.003467 * (OL^4.6)	FL = -37.34 + 27.447 * OL	Observatoire Pelagis
		Gadus morhua / *Pollachius* spp. / *Melanogrammus aeglefinus*	OL	FM = 0.01192 * (OL^4.205)	FL = 13.2 * (OL^1.329)	Härkonen (1986)
	Ammodytidae	Und. Sandeels	OL	FM = 1.083343 * (OL^2.446703)	FL = -4.024 + 56.84 * OL	FM: Brown and Pierce (1998)<br>FL: Härkonen (1986)
	Moronidea	*Dicentrarchus labrax*	OL	FM = 0.0312 * (OL^3.7022)	FL = 39.352 * OL - 172.18	Observatoire Pelagis
	Mugilidae	Und. Mullets	OL	FM = 0.0107 * ((FL/10)^2.948)	FL = (-4.31 + 4.71 * OL) * 10	FM: Observatoire Pelagis<br>FL: Leopold et al. (2001)
Pelagic fish	Clupeidea	*Clupea harengus*	OW	FM = 3.3994 * (OW^3.798)	FL = -49.294 + 132.44 * OW	Lundström et al. (2007)
		Sardina pilchardus	OW	FM = (0.22 * FL / 10)^3	FL = (16.88 * OW - 5.96) * 10	Leopold et al. (2001)
		Sprattus sprattus	OL	FM = (0.18 * FL / 10)^3.5	FL = (11.92 * OW - 1.41) * 10	Leopold et al. (2001)
	Belonidae	*Belone belone*	OL	FM = (0.09 * FL)^3.28	FL = 10.62 * OL + 10.38	Leopold et al. (2001)
	Carangidae	*Trachurus trachurus*	OL	FM = (0.67 * OL)^2.98	FL = -0.9 + 3.29 * OL	Härkonen (1986)
Pelagic squids	Loliginidae	*Loligo* spp.	LRL	FM = exp(2.12 + 2.91 * ln(LRL))	FL = -42.22 + 84.274 * LRL	Clarke (1986)

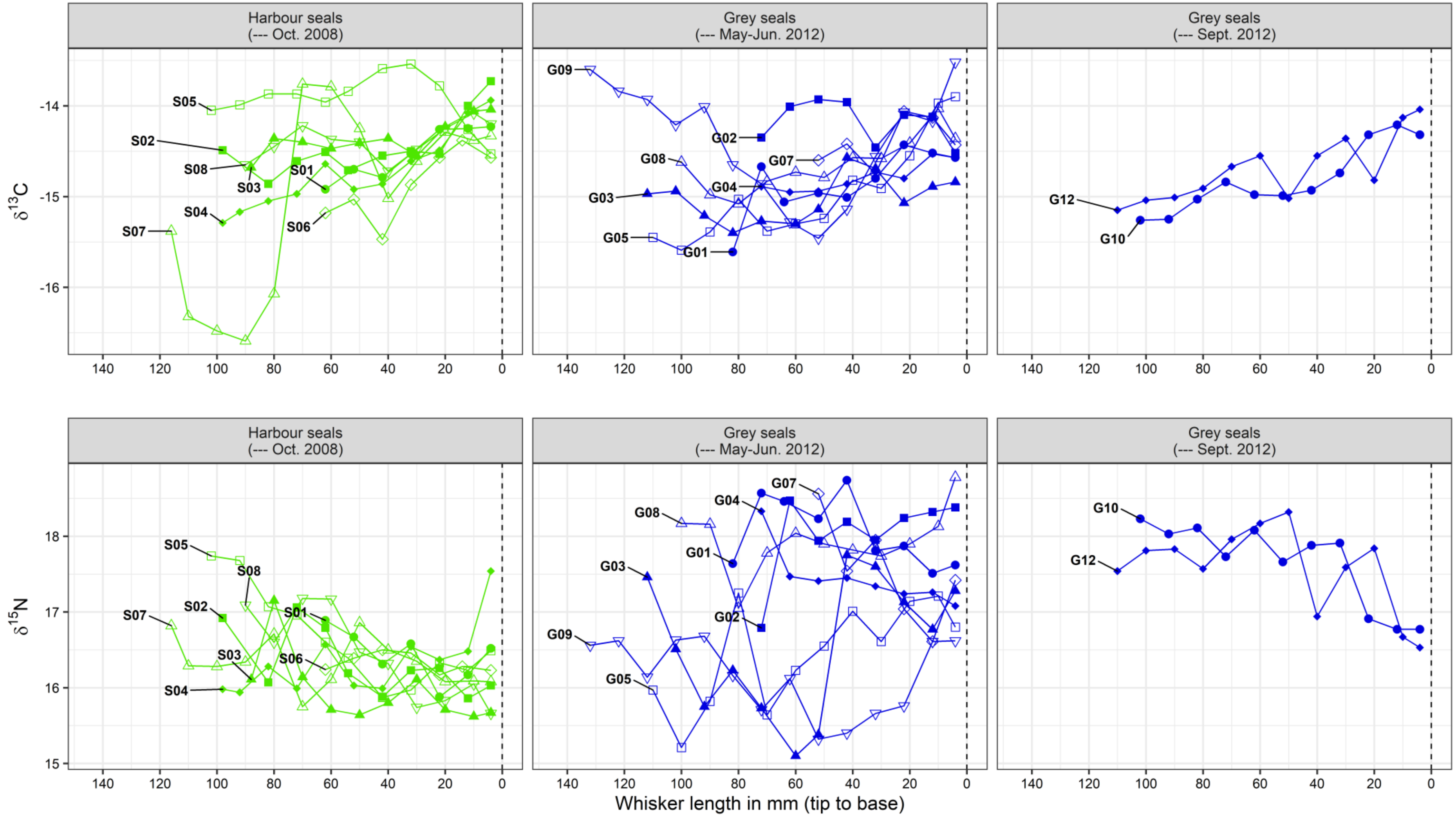

Appendix 2 Raw δ^{13}C and δ^{15}N stable isotope values measured along the whiskers of the eight harbour seals and ten grey seals captured in the Baie de Somme in 2008 and 2012 respectively. Data are ordered by distance to the base of the whisker (0 mm, indicated by dotted vertical lines) from tip to base, i.e. from the oldest to the newest part of the whisker growth.

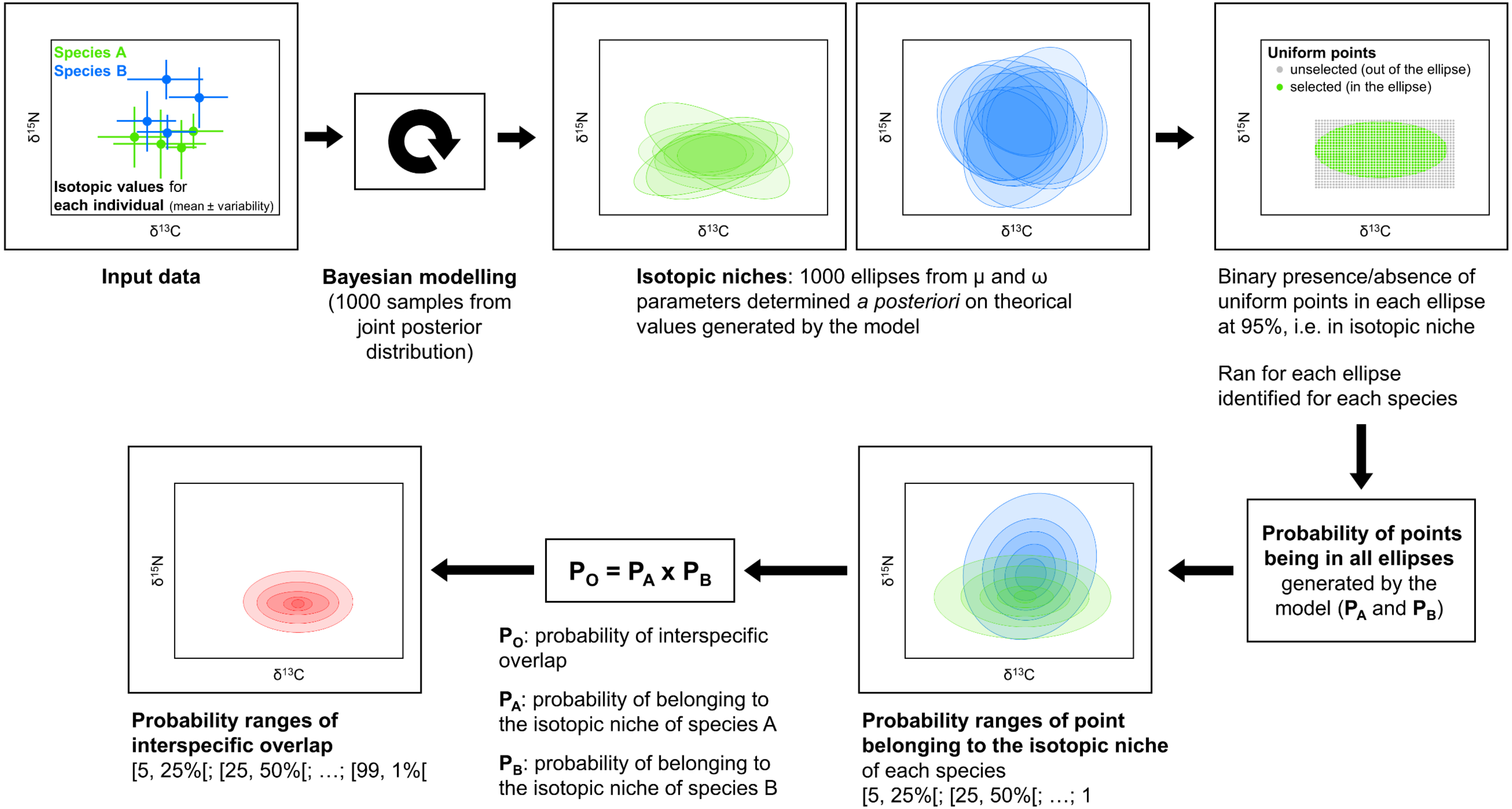

Appendix 3 Steps to identify isotopic niches for the two species using the Bayesian model developed in this study (1,000 ellipses at 95% for two species, in green and blue) and to characterise probability ranges of being in isotopic niches (range ellipses in green and blue) and probability ranges of interspecific overlap between both species (range ellipses in red).

Appendix 4 Details of harbour seal prey content found in scats collected in the Baie de Somme between 2002 and 2019. Of the 196 scats collected, 193 included at least one measurable prey, i.e. diagnostic hard part. Results are classified by prey or group of prey. Occurrence: number (Nscats) and percentage of scats containing this prey type. Nprey: number of prey individuals. CI95%: confidence interval at 95%. Und.: undetermined species.

**Functional group**	**Family**	**Prey**	**Measu-rable prey**	**Occurrence**		**Abundance**			**Prey relative length (mm)**	**Reconstructed prey mass (g)**	**Biomass (kg)**		
				Nscats	%	Nprey	%N	CI95%	Mean ± SD	Mean ± SD	Mass	%Total mass	CI95%
Small benthic flatfish (relative length < 200 mm) and Large benthic flatfish (relative length ≥ 200 mm)	Pleuronectidea	*Pleuronectes platessa* / *Platichthys flesus*	1	140	71.4	1159	17.5	13.3–22.4	160 ± 65	57 ± 1	65.7	25.6	21–31.3
	Soleidae	*Solea solea* / *Pegusa lascaris*	1	119	60.7	1556	23.5	17.1–30.4	172 ± 52	59 ± 65	92.4	36.0	28.9–42.9
		Buglossidium luteum / *Microchirus variegatus*	1	102	52.0	2370	35.8	27.7–43.6	138 ± 30	25 ± 16	60.0	23.4	17.8–29.6
	Bothidea	Und. Bothidea	1	56	28.6	205	3.1	2.2–4.1	107 ± 17	18 ± 8	3.7	1.4	1–1.9
	Scophthalmidae	*Scophtalmus* spp.	1	3	1.5	5	0.1	0–0.2	121 ± 24	26 ± 15	0.1	0.1	0–0.1
		Phrynorhombus norvegicus	1	1	0.5	1	0.0	0–0.1	104	15	0.0	0.0	0–0
		Und. Flatfish	0	4	2.0	6	0.1	0–0.2	–	–	–	–	–
Benthic non-flatfish	Gobidae	Und. Gobidae	1	30	15.3	274	4.1	0.8–9.9	64 ± 11	2 ± 1	0.6	0.2	0–0.6
	Trachinidae	*Trachinus draco*	1	10	5.1	86	1.3	0.2–3	159 ± 23	52 ± 23	4.5	1.7	0.2–4
	Callionymidae	*Callionymus lyra*	1	60	30.6	659	10.0	6–15.7	138 ± 32	21 ± 16	13.7	5.3	2.6–8.9
	Triglidae	Und. Triglidae	1	1	0.5	1	0.0	0–0.1	116	33	0.0	0.0	0–0
Demersal fish	Gadidae	*Merlangius merlangus*	1	5	2.6	9	0.1	0–0.3	187 ± 38	54 ± 34	0.5	0.2	0–0.4
		Trisopterus spp.	1	2	1.0	4	0.1	0–0.2	176 ± 68	72 ± 46	0.3	0.1	0–0.4
		Und. Gadidae	0	1	0.5	1	0.0	0–0.1	–	–	–	–	–
	Ammodytidae	Und. Sandeels	1	9	4.6	203	3.1	0.6–6.7	157 ± 24	14 ± 5	2.9	1.1	0.1–2.7
	Mugilidae	Und. Mullets	1	7	3.6	8	0.1	0–0.2	444 ± 54	802 ± 248	6.4	2.5	0.8–4.8
Pelagic fish	Clupeidea	*Clupea harengus*	1	2	1.0	3	0.0	0–0.1	114 ± 62	15 ± 17	0.0	0.0	0–0.1
		Sardina pilchardus	1	19	9.7	35	0.5	0.3–0.9	227 ± 32	131 ± 54	4.6	1.8	0.8–3.1
		Sprattus sprattus	1	2	1.0	14	0.2	0–0.7	137 ± 22	26 ± 14	0.4	0.1	0–0.4
	Belonidae	*Belone belone*	1	3	1.5	5	0.1	0–0.2	45 ± 3	101 ± 21	0.5	0.2	0–0.5
	Carangidae	*Trachurus trachurus*	1	1	0.5	1	0.0	0–0.1	6	2	0.0	0.0	0–0
Unidentified fish		Und. Fish	0	11	5.6	14	0.2	0.1–0.4	–	–	–	–	–

Appendix 5 Details of grey seal prey content found in scats collected in the Baie de Somme between 2017 and 2019. Of the 126 scats collected, 77 included at least one measurable prey, i.e. diagnostic hard part. Results are classified by prey or group of prey. Occurrence: number (Nscats) and percentage of scats containing this prey type. Nprey: number of prey individuals. CI95%: confidence interval at 95%. Und.: undetermined species.

**Functional group**	**Family**	**Prey**	**Measu-rable prey**	**Occurrence**		**Abundance**			**Prey relative length (mm)**	**Reconstructed prey mass (g)**	**Biomass (kg)**		
				Nscats	%	Nprey	%N	CI95%	Mean ± SD	Mean ± SD	Mass	%Total mass	CI95%
Small benthic flatfish (relative length < 200 mm) and Large benthic flatfish (relative length ≥ 200 mm)	Pleuronectidea	*Pleuronectes platessa* / *Platichthys flesus*	1	25	19.8	136	12.3	6.5–18.3	221 ± 74	146 ± 2	19.9	32.2	18.2–45.2
	Soleidae	*Solea solea* / *Pegusa lascaris*	1	33	26.2	161	14.6	8.9–21.6	189 ± 53	77 ± 2	12.4	20.1	13.5–27.6
		Buglossidium luteum / *Microchirus variegatus*	1	8	6.3	36	3.3	0.9–6.5	148 ± 33	32 ± 6	1.1	1.9	0.6–3.4
	Bothidea	Und. Bothidea	1	4	3.2	5	0.5	0.1–0.9	83 ± 19	9 ± 2	0.0	0.1	0–0.2
		Und. Flatfish	0	6	4.8	17	1.5	0.2–4.1	–	–	–	–	–
Benthic non-flatfish	Gobidae	Und. Gobidae	1	1	0.8	1	0.1	0–0.3	48	1	0.0	0.0	0-0
	Trachinidae	Und. Trachinidae	0	1	0.8	1	0.1	0–0.4	–	–	–	–	–
Demersal fish	Gadidae	*Merlangius merlangus*	1	19	15.1	72	6.5	3–11.4	185 ± 48	57 ± 1	4.1	6.7	3–11.5
		Trisopterus spp.	1	17	13.5	132	11.9	4.6–21.6	152 ± 49	40 ± 0	5.3	8.6	4.6–13.4
		Gadus morhua / *Pollachius* spp. / *Melanogrammus aeglefinus*	1	1	0.8	2	0.2	0–0.6	287 ± 38	214 ± 128	0.4	0.7	0–2.2
		Und. Gadidae	0	1	0.8	1	0.1	0–0.3	–	–	–	–	–
	Ammodytidae	*Und. Sandeels*	1	7	5.6	9	0.8	0.3–1.7	159 ± 62	18 ± 2	0.2	0.3	0–0.7
	Moronidea	*Dicentrarchus labrax*	1	2	1.6	3	0.3	0–0.7	229 ± 68	191 ± 61	0.6	0.9	0–2.7
Pelagic fish	Clupeidea	*Clupea harengus*	1	32	25.4	393	35.5	17.7–51.6	152 ± 66	19 ± 2	7.6	12.3	5.2–23.5
		Sprattus sprattus	1	2	1.6	18	1.6	0–4.3	91 ± 21	7 ± 1	0.1	0.2	0–0.6
	Carangidae	*Trachurus trachurus*	1	1	0.8	2	0.2	0–0.7	13 ± 2	23 ± 16	0.0	0.1	0–0.3
Pelagic squids	Loliginidae	*Loligo* spp.	1	9	7.1	29	2.6	0.5–5.8	253 ± 330	342 ± 150	9.9	16.1	4–33.9
Cephalopods		Und. Cephalopods	0	1	0.8	1	0.1	0–0.3	–	–	–	–	–
Cartilaginous fish		Und. Chondrichthyans	0	1	0.8	1	0.1	0–0.3	–	–	–	–	–
Unidentified fish		Und. Fish	0	56	44.4	86	7.8	4.8–13.2	–	–	–	–	–

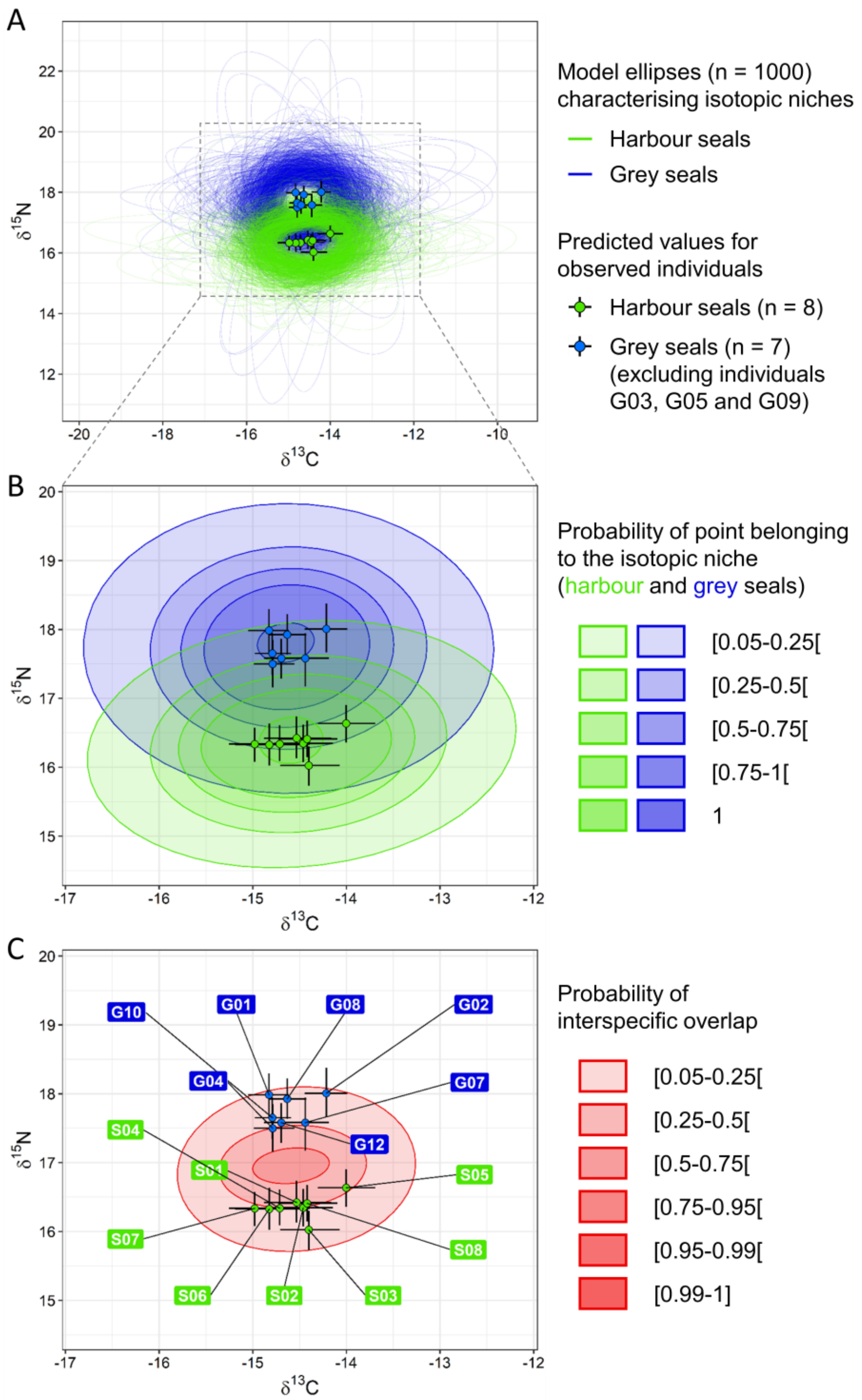

Appendix 6 Isotopic niches of harbour and grey seals estimated using Bayesian modelling run on $\delta^{13}C$ and $\delta^{15}N$ stable isotope ratios measured along a whisker of eight harbour seal and seven grey seal individuals (test realised by excluding the data of three grey seal individuals: G03, G05 and G09). **A**: Isotopic niches characterised by standard ellipses at a 95% confidence interval for harbour seals (green) and grey seals (blue). **B**: Ranges of probability for isotopic niches of harbour and grey seals. **C**: Ranges of probability of interspecific isotopic niche overlap. Each probability range in B and C was characterised by ellipses at 95% around uniform points describing this probability (see Appendix 3). Points in **A**, **B** and **C** are averages of predicted isotopic values for observed harbour and grey seal individuals, and confidence intervals are given at 95%.
